# Prognostic Impact of [18F]Fluorothymidine and [18F]Fluoro-D-Glucose Baseline Uptakes in Patients with Lung Cancer Treated First-Line with Erlotinib

**DOI:** 10.1371/journal.pone.0053081

**Published:** 2013-01-04

**Authors:** Matthias Scheffler, Thomas Zander, Lucia Nogova, Carsten Kobe, Deniz Kahraman, Markus Dietlein, Irini Papachristou, Lukas Heukamp, Reinhard Büttner, Ron Boellaard, Adriaan A. Lammertsma, Silvia Querings, Erich Stoelben, Walburga Engel-Riedel, Bernd Neumaier, Jürgen Wolf

**Affiliations:** 1 Department I for Internal Medicine, University Hospital of Cologne, Cologne, Germany; 2 Center for Integrated Oncology Köln Bonn, Cologne, Germany; 3 Clinic for Nuclear Medicine, University Hospital of Cologne, Cologne, Germany; 4 Institute of Pathology, University Hospital Cologne, Cologne, Germany; 5 Department of Nuclear Medicine and PET Research, VU University Medical Center Amsterdam, Amsterdam, The Netherlands; 6 Max-Planck Institute for Neurological Research, Cologne, Germany; 7 Lung Clinic Merheim, Hospital of Cologne, Cologne, Germany; Stanford University Medical Center, Division of Nuclear Medicine, United States of America

## Abstract

3′-deoxy-3′-[^18^F]fluoro-L-thymidine (FLT) and 2′-deoxy-2′-[^18^F]fluoro-D-glucose (FDG) are used to visualize proliferative and metabolic activity of tumors. In this study we aimed at evaluating the prognostic value of FLT and FDG uptake measured by positron emission tomography (PET) in patients with metastatic non-small cell lung cancer (NSCLC) prior to systemic therapy with erlotinib. FLT and FDG maximum standardized uptake (SUVmax) values per patient were analyzed in 40 chemotherapy naive patients with advanced NSCLC (stage IV) before treatment with erlotinib. Prior therapy median SUVmax was 6.6 for FDG and 3.0 for FLT, respectively. In univariate analysis, patients with an FDG SUVmax <6.6 had a significantly better overall survival (16.3 months [95% confidence interval [CI] 7.1–25.4 months]) compared to patients with an FDG SUVmax ≥6.6 (3.1 months [95% CI 0.6–5.5 months]) (p<0.001, log rank). Similarly, low FLT uptake (SUVmax <3.0) was associated with significantly longer survival (10.3 months (0–23.3 months, 95% CI) compared to high FLT uptake (3.4 months (0–8.1 months, 95% CI) (p = 0.027). The independent prognostic value of baseline FDG uptake was demonstrated in multivariate analysis (p = 0.05, Cox regression). These data suggest that baseline SUVmax values for both FDG and FLT PET might be further developed as markers for prognostic stratification of patients in advanced NSCLC treated with tyrosine kinase inhibitors (TKI) directed against the epidermal growth factor receptor (EGFR).

**Trial Registration:**

Clinicaltrials.gov, Identifier: NCT00568841

## Introduction

Prognostic factors may help to understand the biological heterogeneity of malignant disease and, ultimately, to develop individualized therapeutic strategies for distinct subgroups. In advanced NSCLC, several pretherapeutic prognostic factors have been identified, among these disease stage and performance state [1], [2], [3]. Increasingly, genetic alterations are identified with prognostic as well as predictive potential concerning the use of molecularly targeted drugs. Activating mutations in the epidermal growth factor receptor (EGFR) for instance indicate a better prognosis independent from therapy as well as a favorable outcome with EGFR tyrosine kinase inhibitor (TKI) therapy [4], [5], [6], [7], [8], [9]. However, molecular analyses are not always feasible due to limitations regarding tissue availability and quality [10]. These problems might be circumvented by noninvasive methods.

Molecular imaging tools gain in importance for assessment of tumor biology with and without therapy. 2′-deoxy-2′-[^18^F]fluorodeoxyglucose (FDG) is by far the most commonly used PET tracer, visualizing glucose metabolism. In early stage NSCLC, reports were ambiguous concerning the prognostic value of preoperative FDG uptake, whereas there was no prognostic value in advanced NSCLC treated with standard chemotherapy [1], [11], [12], [13]. In two recent trials, FDG was superior to 3′-Deoxy-3′-[^18^F]fluorothymidine (FLT) in early predicting response and nonprogression in NSCLC patients treated with erlotinib [14], [15]. The use of FDG as a tool for early response prediction was also confirmed in patients with advanced NSCLC undergoing chemoradiotherapy [16]. In esophageal cancer, FDG baseline activity is predictive for response [17]. In BRAF-mutated advanced melanoma treated with vemurafenib, there was a trend for longer profession-free survival (PFS) in patients with low metabolic disease assessed by FDG-PET [18].

FLT is a noninvasive marker of proliferation and has been shown to correlate with Ki-67 expression in NSCLC [19], [20], [21], [22]. Proliferative activity has been discussed to have a negative impact on survival [23], [24], [25], although the definitive relationship remains unclear [26]. In NSCLC, the ability of FLT as a PET tracer to early visualize G1-cell cycle arrest and induction of apoptosis was demonstrated in xenotransplanted cell lines sensitive to erlotinib, and early reduction of FLT uptake predicted response in patients treated with gefitinib and erlotinib [27], [28], [29]. In patients with aggressive B-cell lymphomas treated with the R-CHOP regimen high baseline FLT uptake is a negative predictor for response [30]. In patients with NPM-ALK-positive lymphomas treated with targeted therapy, FLT-PET was superior to FDG-PET for very early response prediction [31].

Based on results of a monocentric clinical trial, we analyzed if already the initial proliferative (FLT) or metabolic (FDG) activity of NSCLC tumors assessed by PET is associated with overall survival irrespective of clinical trial protocol adherence, follow-up treatments or very early progression and how EGFR mutational status and Ki-67 immunohistochemistry as well as clinical parameters contribute to these findings.

## Patients and Methods

### Patients

Between September 2007 and September 2009, patients with cytologically or histologically confirmed metastatic NSCLC (International Union Against Cancer [UICC] stage IV) and without prior systemic treatment had undergone one FDG-PET and one FLT-PET prior to systemic therapy within the screening program of the ERLOPET trial (NCT00568841), which was approved by the institutional review board, the local ethics committee and the respective federal and state authorities, including the German Authority for Radiation Safety. 34 of the 40 patients presented here could be analyzed in the ERLOPET trial. The protocol for this trial and supporting CONSORT checklist are available as supporting information; see Checklist S1 and Protocol S1. All patients gave written informed consent. As part of the screening process, the patients had to be at least 18 years old, with an Eastern Cooperative Oncology Group (ECOG) performance state ≤2, neither decompensated liver nor heart failure, a serum creatinine level <1.7 mg/dL, and normal blood glucose levels (<120 mg/dl). Patients with brain metastases requiring further local treatment were not excluded. For this analysis all patients that underwent baseline PET were evaluated irrespective of later trial exclusion e.g. due to stop of medication.

### Treatment

All patients were intended to start with erlotinib 150 mg/d for at least six weeks or until disease progression. In case of progression, a platinum-based combination therapy was the recommended treatment option. After progress to platinum-based chemotherapy patients were treated either with chemotherapy (pemetrexed, docetaxel, gemcitabine or vinorelbine) or targeted therapy (sorafenib plus everolimus within a clinical trial (NCT00933777) or afatinib within a compassionate use program). The mean number of treatment regimens was 2 (range, 0 to 5), with the following regimens used: carboplatin/paclitaxel +/−bevacizumab, cisplatin/vinorelbine +/− cetuximab, pemetrexed, pemetrexed maintenance, docetaxel, gemcitabine, oral vinorelbine, sorafenib/everolimus (one patient in forth-line setting,) and afatinib. Early palliative care was performed as described [32]. Radiation therapy was performed whenever indicated: 9 patients (23%) were pretreated with radiation therapy (5 patients receiving either whole-brain radiation or stereotactic intervention due to brain metastases, 2 patients with local treatment of symptomatic bone metastases, 2 patients with mediastinal/lung radiation due to local complications), 7 patients (18%) received radiation therapy while being treated with erlotinib (2 whole-brain radiation, 5 bone metastases), and 5 patients were irradiated after stop of erlotinib treatment and change to an alternative systemic therapy (1 whole-brain radiation, 2 local irradiation, 2 bone metastases). One patient died before start of therapy. 13 patients [32.5%] had brain metastases at baseline. In general, radiation therapy was performed in 21 (52.5%) patients, whereof 9 patients (22.5%) had to start radiation therapy due to local complications (brain, bones) before administration of systemic therapy. Some of the details are also shown in **table 1**.

**Table 1:** Patient characteristics.

Characteristics		Number (%)
All patients		40 (100)
Gender	female	21 (53)
	male	19 (47)
Histology	Adeno/BAC	34 (85)
	others	6 (15)
ECOG	0	17 (42.5)
	1	17 (42.5)
	2	6 (15)
EGFR mutation detected	yes	5 (12.5)
	no	35 (87.5)
Brain metastases	yes	13 (32.5)
	no	27 (67.5)
Local radiation	yes	21 (52.5)
	no	19 (47.5)

### Response evaluation

Response was assessed using the Response Evaluation Criteria in Solid Tumors (RECIST) Version 1.0 [33]. The first computed tomography (CT) scan was performed after six weeks of treatment. Follow-up CT scans were performed every 12 weeks or in case of clinically suspected progression. A 16-slice multidetector CT scanner (Brilliance 16, Philips Medical Systems, Eindhoven, the Netherlands) was used.

### Clinical parameters

The following parameters were assessed prior to therapy start to evaluate their impact on overall survival: age (dichotomized), ECOG (0–2), EGFR mutational status, histology (adeno/bronchiolo alveolar carcinoma histology vs non-adeno/BAC), gender.

### PET-Imaging

**Figure 1:**
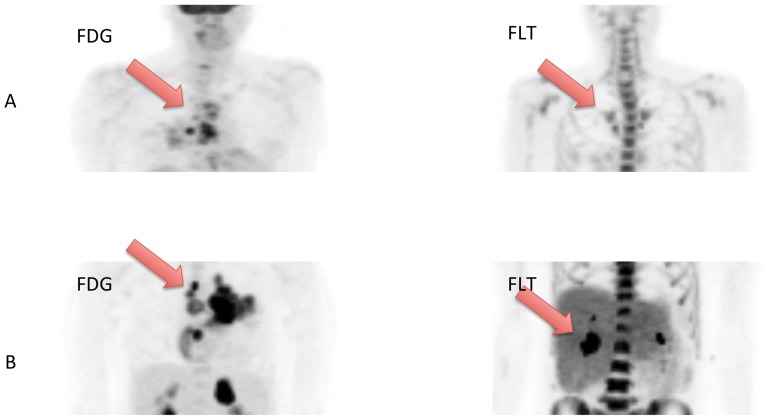
Example of two patients with low and high baseline uptake of FDG and FLT. The patient shown in figure A with low uptake is a 66-year old female patient who had an overall survival of 21.3 months, whereas the patient in B with a high uptake is a 56-year old female patient with an overall survival of only 1.5 months. In both cases, the respective most active lesion was chosen for assessment.

FLT-PET and FDG-PET were performed before treatment administration. Both tracers were synthesized as described before [34]. The images were obtained using an ECAT EXACT 47 (Siemens, Erlangen, Germany). Patients had to be fasting for at least 6 hours. 60 minutes after injection of 300 MBq FLT or 370 MBq FDG, the PET acquisition started. The attenuation-corrected scan trajectory covered 90 cm (6 bed positions: 5 min emission, 3 min transmission). All scans were corrected for decay, dead time, scatter and randoms, and reconstructed by ordered subset expectation maximization. The same protocol for acquisition and the same software for reconstruction were used. The maximum standardized uptake value (SUVmax) normalized to body weight was assessed using the voxel with the maximum uptake on reconstructed PET images without additional rebinning, resampling, or smoothing. Up to five lesions with the highest SUVmax uptakes were coregistered. The highest SUVmax for the respective tracer, not necessarily the same lesions, were taken into analysis (**figure 1**).

### Molecular analysis

Tumor material from the initial diagnosis of NSCLC was analysed for EGFR mutational status. If there was still tumor material left after the mutational analyses, Ki-67 immunohistochemistry staining was performed.

EGFR mutational status was assessed as recently reported, using PCR and dideoxy sequencing, pyrosequencing and massively parallel sequencing analyses dependent on tissue quality and amount of tumor cells . Ki-67 immunohistochemistry staining was performed using standard techniques.

### Statistical analysis

The trial was powered for its primary objective [14]. Time-to-event analyses were exploratorily assessed. Overall survival (OS) and PFS were defined as the time from start of treatment until the respective event and analysed using Kaplan-Meier estimates and log rank tests for univariate analysis. For continuous parameters, the median was chosen to divide the cohort in order to build homogenous subgroups of patients. Parameters showing statistical significance (p≤0.05) in univariate analysis were included in a Cox regression for multivariate analysis. FDG- and FLT-SUVmax values and age were dichotomized by their median to achieve homogenous subgroups for Kaplan-Meier estimates. For correlation analysis, Pearson's correlations were used. For response analysis, receiver-operator-characteristics (ROC) curves were created. Students T test was performed were applicable.

## Results

### Patients

40 patients received both FLT-PET and FDG-PET prior to treatment start. At data cut-off (May 18^th^, 2011), 4 patients (10%) were still alive, with a median follow-up of 25.6 months (range, 23.4–34.0). 3 of these patients are women with adenocarcinoma, one with a detected EGFR mutation. The fourth is a male patient with squamous-cell carcinoma. 34 patients had adenocarcinoma/BAC histology (85%). The mean age was 62.5 years (range, 38–78 years). 31 patients (77.5%) had tumor tissue available (formalin-fixed paraffin-embedded biopsies, stained cytospins) for EGFR mutational analysis. Thereafter, 18 tissue samples remained for Ki-67 staining. Five patients (12.5%) had sensitizing EGFR mutations (4 deletions in exon 19, one L858R). Patient characteristics are shown in **table 1**. Six patients (15%) had an ECOG 2 performance state, 17 patients (42.5%) ECOG 1. One patient died immediately (3 days) after PET scans due to deterioration of an underlying pneumonia. For this patient, time from the PET scans until death was calculated for time-to-event analyses.

5 patients (12.5%) responded to erlotinib, and additional 7 patients (17.5%) had a stable disease lasting for at least 18 weeks. 28 patients (70%) had either documented progressive-disease (PD) in the first CT scan after six weeks of treatment or clinical progression before, including rapid deaths.

### Clinical parameters

Of the clinical parameters tested, only the division of patients by the median of age (62.5 years) led to significantly different groups regarding median OS (mOS), favoring older patients (14.9 months [95% CI, 3.0–26.7 months] vs 3.4 months [0–6.8 months, 95% CI], p = 0.030). Presence of an activating EGFR mutation status had no significant impact on survival in our cohort (EGFR mut: mOS of 21.3 months (9.9–32.8 months; EGFR wt: 4.8 months (2.3–7.3); log-rank (p = 0.087)). Similarly, OS differences did not reach significance in log rank for gender (mOS in women 10.3 months (2.3–18.2) compared to 4.8 months (2.6–7.0) in men; p = 0.214, log rank), histology (mOS in non-adeno/BAC 0.9 months (0–4.2) compared with 5.4 months in adenocarcinoma (0–12.4); p = 0.467, log rank) and performance state (ECOG 0 = 14.9 (7.0–22.8); ECOG 1 = 1.4 (2.6–8.1) ECOG 3 = 0.3 (0.1–1.2); p = 0.141, log rank).

In contrast, EGFR mutational status (p = 0.008, log rank), age (p = 0.009, log rank, in favor of the older patients), and ECOG performance status (p = 0.004, log rank) were significantly associated with a longer progression free survival.

### PET analysis

The majority of patients (n = 36, 90%) underwent PET scans on consecutive days. In one patient, FDG-PET scan was conducted two days prior to FLT-PET scan. In another patient, FDG-PET was performed four days before FLT-PET, while in two patients, FLT-PET was performed three days prior to FDG-PET. **Table 2** shows the individual patient characteristics and PET results.

**Table 2:** Individual patient characteristics and PET results.

Pat-ID	Gender	Age	Histology	SUVmax (FDG)	Tissue (SUVmax FDG)	SUVmax (FLT)	Tissue (SUVmax FLT)	Differernt lesions
01-01	f	53	Adeno	7,9	lung	2,6	lymph node	yes
01-02	m	69	Adeno	7,5	adrenal gland	3,3	adrenal gland	no
01-03	m	58	Adeno	7,8	lung	3,3	adrenal gland	yes
01-04	f	69	Adeno	13,0	lung	2,5	lung	no
01-05	m	67	SCC	11,9	lung	3,3	lung	no
01-06	m	69	SCC	8,3	lung	2,9	lung	no
01-07	f	69	SCC	5,7	lung	3,9	lymph node	yes
01-08	m	64	Adeno	4,0	pleura	2,5	lung	yes
01-09	m	55	Adeno	9,3	pleura	5,5	bone	yes
01-10	m	38	SCC	5,5	lymph node	4,0	thoracic wall	yes
01-11	m	51	Adeno	3,4	bone	1,3	bone	no
01-12	f	53	Adeno	1,6	adrenal gland	1,3	adrenal gland	no
01-13	m	55	Adeno	6,6	thoracic wall	4,6	pleura	yes
01-14	m	60	Adeno	4,5	lung	2,5	lung	no
01-15	m	53	Adeno	7,0	lymph node	3,4	lymph node	no
01-16	f	45	Large cell	6,6	bone	1,9	bone	no
01-17	f	72	Adeno	5,7	lung	5,5	lung	no
01-18	f	78	BAC	2,0	lung	1,8	lung	no
01-19	m	67	BAC	3,9	lung	1,6	lung	no
01-20	m	60	BAC	4,0	bone	1,5	bone	no
01-21	f	57	Adeno	11,0	adrenal gland	5,5	lung	yes
01-22	f	61	Adeno	5,8	thoracic wall	5,0	bone	yes
01-23	f	55	Adeno	2,8	lung	2,0	lymph node	yes
01-24	m	67	Adeno	6,3	lung	2,3	lung	no
01-25	f	61	Adeno	1,9	pleura	1,4	pleura	no
01-26	m	63	Adeno	8,0	lung	5,0	lung	no
01-27	f	66	Adeno	5,1	lymph node	3,0	lymph node	no
01-28	f	71	Adeno	7,2	lymph node	2,7	lymph node	no
01-29	f	57	SCC	3,9	lymph node	4,1	lymph node	yes
01-30	f	73	Adeno	2,5	lymph node	1,5	lymph node	no
01-31	f	68	Adeno	3,6	lung	1,3	lung	no
01-32	f	48	Adeno	9,1	lung	5,0	lung	no
01-33	m	71	SCC	3,0	lymph node	3,0	lymph node	no
01-34	m	75	Adeno	13,0	lung	3,0	lung	no
01-35	m	77	Adeno	8,5	bone	3,0	lymph node	yes
01-36	f	72	Adeno	13,3	lymph node	5,3	lymph node	no
01-37	f	56	Adeno	9,7	lung	2,8	lung	no
01-38	f	58	Adeno	12,3	bone	2,2	bone	no
01-39	f	78	Adeno	7,1	lung	3,0	lung	no
01-40	m	62	BAC	7,4	lung	4,8	lung	yes

By analysing up to 5 lesions per patient we assessed the lesions with the highest SUVmax values in both FDG and FLT. In 13 patients (32.5%) the lesions differed between FDG and FLT (see **table 2**).

A total of 157 lesions (mean, 3.9 lesions per patient) detected with FDG-PET were analyzed. Of these 157 lesions, 134 (85.4%) showed activity in FLT-PET, too (mean, 3.4 lesions per patient). The 23 discrepant lesions, which did not show activity in FLT-PET, were located within the following tissues: bone (n = 8, 34.8%), lymph nodes (n = 6, 26.1%), liver (n = 3, 13.0%), thoracic wall (n = 3, 13.0%), pleura (n = 2) and adrenal gland (n = 1).

### Association of baseline SUVmax and overall survival (OS)

All patients underwent both FDG- and FLT-PET 0–9 days prior to start of therapy.

**Figure 2:**
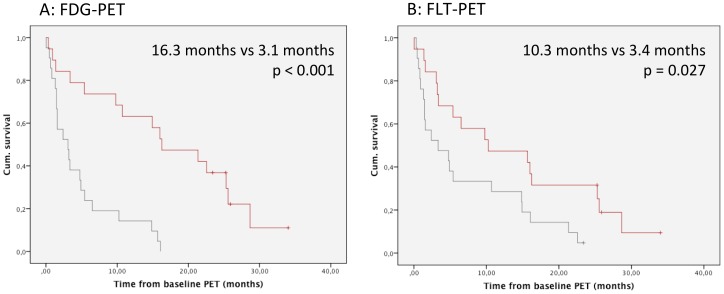
Kaplan Meier curves showing overall survival depending on SUVmax values. A) Overall survival of patients with high (>6.7; grey) or low (<6.7; red) baseline SUVmax in FDG-PET. (B) Overall survival of patients with high (>3; grey) or low (<3; red) baseline SUVmax in FLT-PET.

No significant differences in mean SUVmax of FLT and FDG could be noted between patients with or without adeno/BAC histology (p = 0.921 for FDG and p = 0.873 for FLT, T-test). The SUVmax values of the most active tumor manifestation for FDG had a mean of 6.7 (5.7–7.7) and a median of 6.6. Taking this value as a cut-off, two groups were built. Patients with low SUVmax (SUV<6.6) demonstrate a significantly longer survival (Hazard ratio [HR] 4.3, [95% CI 1.9–9.6]; p<0.001) of 16.3 months (7.1–25.4, n = 19) when compared to patients with high SUVmax (3.1 months, 0.6–5.5, n = 21)(**figure 2A**).

For FLT, SUVmax had a mean value of 3.1 (2.7–3.6), confirming the reported ratio of FDG/FLT [20], [35]. The median value of SUVmax for FLT was 3.0. Patients with an SUVmax <3.0 (n = 19) had a mOS of 10.3 months (0–23.3), and patients with an SUVmax ≥3.0 (n = 21) had a mOS of 3.4 months (0–8.1)(HR 2.2 [95% CI 1.1–4.4]; p = 0.027) (**figure 2B**).

The SUVmax values of FDG and FLT were significantly correlated (Pearson correlation coefficient 0.468, p = 0.002). Baseline FDG-PET was shown to be an independent prognostic factor in a multivariate cox regression model including FLT, FDG and age as categorial (p = 0.05) or continous (p = 0.001) variables. Even when adding EGFR mutation into the model, baseline FDG SUVmax remained an independent prognostic factor (p = 0.002) Also in the group of patients without detected EGFR mutation low SUVmax (<6.6) in FDG-PET was associated with a significantly better overall survival (10.7 months mOS (0.7–20.8 months) vs 3.1 (0.8–5.4 months)(p = 0.002). No such association was observed for FLT-PET in this group (p = 0.077).

We also investigated if one of the parameters (FDG-/FLT-uptake) acts as a prognostic marker in the absence of the other. For FDG SUVmax in Cox regression with age, a p-value<0.001 was reached (age, p = 0.016). For FLT SUVmax, both FLT and age reached significance (p = 0.017 and p = 0.018).

### Association of baseline SUVmax and PFS and response

The mean SUVmax values of EGFR-mutated tumors for both FDG and FLT were significantly lower than in tumors not harboring a mutation (p = 0.033 for FDG and p = 0.027 for FLT, T-test). Consequently, we observed an association between SUVmax in baseline FDG- and FLT-PET and response to erlotinib treatment (AUC of 0.79 (FDG) and 0.78 (FLT) (p = 0.035 for FDG, p = 0.043 for FLT). This higher probability of response in patients with low FDG and FLT uptake did not transfer into a prolonged PFS.

### Ki-67 staining and correlation with PET and EGFR mutational status

Ki-67 was stained in 18 samples to independently assess the proliferative status of these tumors. Overall, no significant correlation between Ki-67 staining and SUVmax of FDG and FLT was found, but patients with low FLT SUVmax values tended to have low Ki-67 activity. Nevertheless, this was not significant (p = 0.168). For FDG, virtually no difference of Ki-67 percentage could be determined in patients with high or low baseline SUVmax (p = 0.936, T-test).

**Figure 3:**
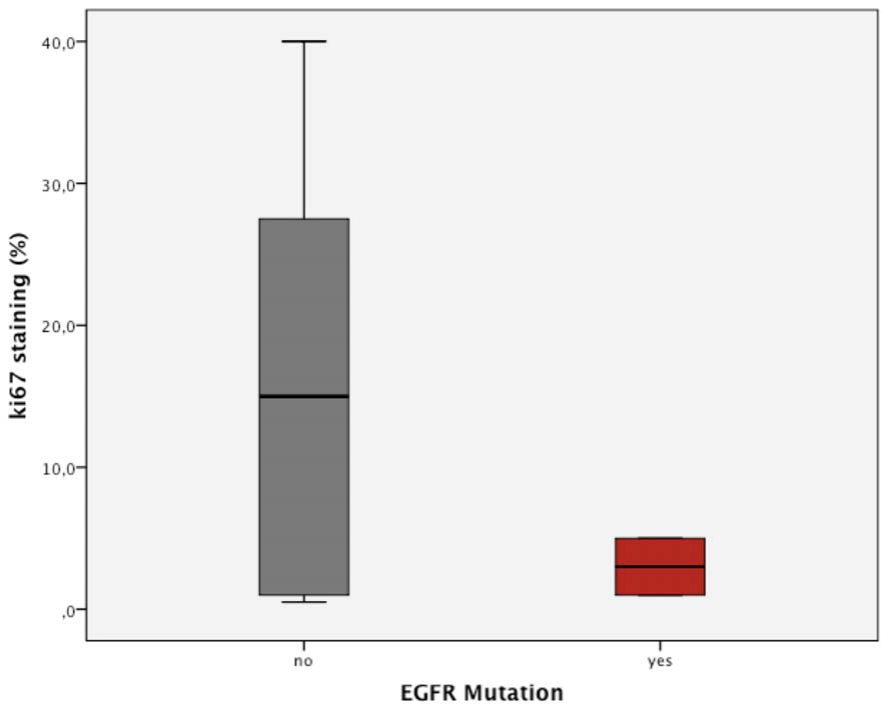
Correlation of the EGFR mutational status with Ki-67 staining in %.

Patients with EGFR mutations showed a significantly lower percentage of Ki-67 positive cells compared to wildtype patients (p = 0.01, T-test) (**figure 3**). Further, the three responding patients had a significantly lower Ki-67 percentage than patients not responding (p = 0.002, T-test).

The median positive staining percentage was 10%. There was no significant difference between patients with a percentage <10% and patients with a percentage ≥10% regarding OS (p = 0.225, log rank), but PFS differed significantly (6.0 [3.8–8.2] vs 1.6 [1.4–1.8] months, p = 0.030, log rank; for the low Ki-67 group).

## Discussion

The aim of this hypothesis generating analysis was to evaluate and compare FDG and FLT baseline activity in the most active tumor manifestations regarding their impact on the prognosis of patients with newly diagnosed advanced NSCLC before start with erlotinib treatment. The baseline uptakes of both tracers are shown to be prognostic in univariate analysis, with FDG being an independent prognostic factor in multivariate analysis as the major finding. These results show that the initial metabolic (FDG) and proliferative (FLT) activity is associated with survival of the patients. Similarly, low metabolic and proliferative activity was associated with a higher probability of response in this group of patients.

Sensitizing EGFR mutations in NSCLC are the strongest predictors of PFS, response and OS under gefitinib and erlotinib therapy [9]. As the five patients with EGFR mutations in our analysis had significantly lower baseline uptakes of both FDG and FLT, it is tempting to speculate that EGFR mutated tumors have lower proliferative activity and that this might contribute to the better prognosis of these patients even if not treated with erlotinib or gefitinib. Similarly, Ki-67 staining demonstrated significantly lower Ki-67 positive cells in EGFR mutated tumors compared to EGFR wildtype tumors as has been reported previously [36]. Nevertheless, even after excluding the five patients with EGFR mutations from the analysis, the baseline FDG uptake remained a strong and independent prognostic factor.

Our results are in line with recent results showing the prognostic value of FDG-PET concerning OS in patients with NSCLC treated with standard chemotherapy and radiotherapy [37]. In contrast, the prognostic significance of FDG-PET was limited for patients with advanced ovarian cancer and in patients with locally advanced rectal cancer receiving neoadjuvant chemoradiotherapy and radical surgery [38], [39] One thus might speculate, that the pretherapeutic tumor glucose metabolism per se is not a strong and independent predictor of overall survival, independent from tumor type and therapeutic modality.

FLT was introduced in cancer imaging as a proliferation-specific marker [40]. In our analysis, FLT was strongly prognostic in univariate analysis, but not in multivariate analysis. Thus, compared to FDG-PET, FLT does not add more specific information regarding prognosis. Whether this is due to the lack of tumor specificity of FLT as recently reported, remains an open question [38], [41], [42]. However, also the prognostic value of the tissue-based proliferation marker Ki-67 has not been established unequivocally so far for advanced NSCLC, most studies available focus on resectable tumors [25], [43], [44]. Surprisingly, in our dataset, there was no significant association between FLT uptake in the hottest lesion and Ki-67 staining in the tumor tissue obtained for diagnosis. This might, however, be due to the fact that in our series in many patients biopsy was not obtained from the lesion with the maximum SUVmax, clearly a limitation of this analysis. In addition, this observation underlines once more again the limited informative value of tissue-based biomarkers requiring invasive biopsy and thus being restricted to one tumor site. A recent meta-analysis describes the difficulties in interpreting findings from clinical trials [22]. Taken together, although FLT-PET seems to be a good tool for response prediction in some tumor entities, its prognostic value remains unclear.

In our analyses we selected up to 5 lesions with the highest activity in FDG- or FLT_PET for evaluation of the prognostic value in concordance with recent studies and recommendations for response prediction [45]. Clearly, this procedure does not allow any conclusion of the total tumor burden, which might be considered a further limitation of this study. On the other hand it is also conceivable to assume that the most active lesion in PET is the prognostically limiting lesion. Finally, we chose this procedure because of its easy-to-access character in clinical settings with the advantages of real-time detection and low inter-observer variability. This consideration also underlied the use of SUVmax and not other PET parameters like SUVpeak, SUVmean or SUVdispersion. We have recently shown that there are no significant differencies concerning the predictive potential of SUVmax and SUVpeak early after initiation of EGFR-therapy [46].

In summary, we show that the identification of the lesion with the highest metabolic activity in FDT-PET has significant prognostic relevance before initiation of erlotinib therapy independent of the EGFR-mutational status. Thus, pretherapeutic metabolic activity might be established in further studies as a risk-stratification tool for clinical trials in advanced NSCLC.

## Supporting Information

Checklist S1 **CONSORT checklist of the trial. Please note that this trial was not randomized.**
(DOC)

Protocol S1 **Trial protocol of the underlying trial of this study.**
(PDF)
