# Supplementary material for: Prognostic Impact of [18F]Fluorothymidine and [18F]Fluoro-D-Glucose Baseline Uptakes in Patients with Lung Cancer Treated First-Line with Erlotinib
Source: PLoS One. 2013 Jan 4;8(1):e53081. doi: 10.1371/journal.pone.0053081 (PMC3537767; doi:10.1371/journal.pone.0053081)
Supplement: Protocol S1 — Trial protocol of the underlying trial of this study. (PDF) [file pone.0053081.s002.pdf]

Clinical Study Protocol

(including second substantial amendment [Amendment 02, 16.11.2009])

ERLOPET

Erlotinib and sequential PET

A Phase II Clinical Trial to Evaluate the Accuracy of FDG-/FLT- PET for Early Prediction of Non-Progression in Patients with Advanced Non Small Cell Lung Cancer (NSCLC) treated with Erlotinib and to Associate PET Findings with Molecular Markers

Lung Cancer Group Cologne (LCGC)

Sponsor: Universität zu Köln

Albertus Magnus Platz

50923 Köln

EudraCT: 2005-005393-73

SponsorNr: ERLOPET_01

V3.01, 16.11.2009

Lung Cancer Group Cologne (LCGC)
Chairman / LKP: Prof. Dr. J. Wolf
University Clinic Cologne
Kerpenerstr. 62
50924 Cologne
LCGC Coordination Center: Marion Schwartzkopff
Department I of Internal Medicine, University Clinic Cologne
Kerpenerstr. 62
50924 Köln
Tel: 0221 / 478-3410
Fax: 0221 / 478-6733
marion.schwartzkopff@uk-koeln.de

0 SUMMARY AND GENERAL INFORMATIONS 8
0.1 Responsible Persons 8
0.2 Declaration by the trial investigators 9
0.3 Protocol synopsis 10
0.4 Flowsheet 13
1 STUDY DESIGN: RATIONALE 15
1.1 Systemic treatment options in NSCLC 15
1.2 EGFR-targeted therapy in NSCLC 15
1.3 Correlation of EGFR/ KRAS mutations and EGFR immunohistochemistry to clinical benefit 16
1.4 FDG- and FLT-PET for assessment of response to treatment 17
2 AIM OF TRIAL 18
3 TRIAL PLAN 19
3.1 Trial design, primary and secondary objectives 19
3.2 Recruitment 19
3.3 Duration of the study 20
3.4 Termination of protocol treatment 20
3.5 Early termination of trial 20
4 ENROLLMENT 20
4.1 Inclusion criteria 20
4.2 Exclusion criteria 21
5 INDIVIDUAL TRIAL PROCEDURE 21
5.1 Diagnosis and allocation to trial 21
5.2 Pseudonymization 22
5.3 Mutation analysis 22
5.4 EGFR immunohistochemistry 22
5.5 Pre-Study Screening (staging examination) 23
5.6 PET 24
5.7 Patient briefing and termination of protocol treatment 25
5.8 Enrollment into the trial 26
5.9 Treatment 27
Erlotinib (Tarceva®) 27
Administration of therapy 28
Side effects 28
Precautions 29
Restaging procedures during and after erlotinib therapy 31
Therapeutic procedure after 6 weeks of erlotinib therapy 32

5.10 Follow-up examinations after end of treatment 32
6 ADVERSE EVENTS 32
6.1 Handling of Safety Parameters 32
Serious Adverse Events 32
Treatment and Follow-up of Adverse Events 33
Follow-up of Abnormal Laboratory Test Values 33
Pregnancy 33
6.2 Definitions 34
6.3 Treatment postponement, dose reduction and termination. 35
6.4 Handling of FDG and FLT safety 36
7 STATISTICS 37
7.1 Statistical formulation of study objectives and design 37
7.2 Analysis and trial termination strategy 38
7.3 Further analyses 39
7.4 Data analysis 40
8 DOCUMENTATION AND MONITORING 41
8.1 Direct access to source data 41
8.2 Quality control and assurance 41
8.3 Monitoring 42
9 ETHICAL MATTERS 43
10 ORGANIZATION 44
10.1 Retrospective protocol amendments 44
10.2 Insurance 44
10.3 Agreement on publication 44
11 REFERENCES 45
12 APPENDIX 51
12.1 Copies of legal documents 51
Declaration of Helsinki 51
Vote of approval by the ethical committee of Universitätsklinikum Köln (original) 55
Confirmation of insurance 56
12.2 Addresses 57
12.3 Definitions 58
Activity index according to the ECOG 58
Stage of disease 59
Response Evaluation Criteria in Solid Tumors (RECIST) Quick Reference 60
Procedures for PET examination 65
Procedures for EGFR and KRAS gene sequencing 65

Quality control 66
Procedure for the performance of computed tomography 66
12.4 Patient's consent 67
Guide to patient briefing 67
Patienteninformation und Einverständniserklärung 70
Einwilligungserklärung 82
Schwangerschaft/Stillen/Kontrazeption 85

Abbreviations

AE	adverse event
AMG	Arzneimittelgesetz (German regulations concerning the production and sale of medicines)
An	anamnesis case report form
γ-GT	γ-glutamyl transferase
AP	alkaline phosphatase
ASCO	American Society for Clinical Oncology
AUC	Area under ROC curve
BAL	bronchoalveolar lavage
BfArM	Bundesinstitut für Arzneimittel und Medizinprodukte (German medicines and medical products authority)
BM	bone marrow
CHD	coronary heart disease
CR	complete remission
CRF	case report form
CRP	C-reactive protein
CRr	CR with residual abnormalities (= CU)
CS	clinical stage
CT	computed tomography
CTC	common toxicity criteria
DCO	diffusion capacity for oxygen
EDTA	ethylene-diamine-tetra-acetic acid
EC	erythrocyte concentrate
ECG	Electrocardiogram
EORTC	European Organization for Research and Treatment of Cancer
ESR	erythrocytes sedimentation rate
F	follow up case report
FA	final (data) analysis
FDG	fluoro-deoxy-glucose
FLT	fluoro-L-thymidine
FFTF	freedom from treatment failure
GCP	good clinical practice
GOT	glutamate oxalacetate transaminase
GPT	glutamate pyruvate transaminase
Hb	Hemoglobin
HR-CT	high resolution computed tomography
IA	interim (data) analysis
ICDO	International Classification on Diseases for Oncology
ICH	International Conference on Harmonization

ICRU	International Commission on Radiation Units and Measurements
ITT	intention to treat
KKSK	Koordinationszentrum für Klinische Studien Köln
LDH	lactate dehydrogenase
LN	lymph node(s)
QoL	quality of life
LV	left ventricle
MDS	myelodysplastic syndrome
MFI	Multidimensional Fatigue Inventory
MTBTI	Molecular Tumor Biology and Tumor Immunology
NC	no change
NCI-CTC	National Cancer Institute Common Toxicity Criteria
NMR	nuclear magnetic resonance
NYHA	New York Heart Association
OS	overall survival
PCO_2	carbon dioxide partial pressure
PCR	polymerase chain reaction
PD	progressive disease
PET	positron emission tomography
PO_2	oxygen partial pressure
PR	partial remission
PS	pathological stage
RE	restaging case report form
REG	registration case report form
RF	risk factor
RFS	relapse free survival
S	staging case report form
SD	stable disease
SAE	serious adverse event
SOPs	standard operating procedures
SUV	standard uptake value
TEE	transesophageal echocardiography
Th	therapy case report form
TSH	thyroid stimulating hormone
UNL	upper normal limit
WHO	World Health Organisation

0 SUMMARY AND GENERAL INFORMATIONS

0.1 Responsible Persons

Sponsor:	University Cologne represented by Prof. Wolf<br>Albertus Magnus Platz, 50923 Köln
LKP :	Prof. Dr. Jürgen Wolf<br>Kerpenerstr. 62, 50924 Cologne, Germany
Cologne, __/__/____	__________________
Co-investigators:	Dr. Thomas Zander, Dr. Lucia Nogová, Dr. Karin Töpelt, Dr. Roman Thomas, Matthias Scheffler, Dr. Andreas Draube, Dr. Martin Weihrauch
PET:	PD Dr. Markus Dietlein, PD Dr. Matthias Schmidt, Dr. Carsten Kobe, Juliane Bachmann
Radiology:	Dr. Christopher Bangard, Prof. Dr. Barbara Krug, Dr. Gesa Eyl
Pathology:	PD Dr. Claudia Wickenhauser
Statistics:	PD Dr. Martin Hellmich
Cologne, __/__/____	__________________
Writing committee:	Dr. Thomas Zander, Dr. Lucia Nogová,<br>Dr. Roman Thomas, Matthias Scheffler
Monitor / Datamanagement:	ZKS Köln
Study committee:	Prof. Dr. Michael Hallek<br>Dr. Andrea Koch<br>Prof. Dr. Klaus Lackner<br>Prof. Dr. Rolf-Peter Müller<br>Prof. Dr. Harald Schicha<br>PD Dr. Paul Schneider<br>Prof. Dr. Joachim Schultze
Data safety monitoring board	Prof. Dr. Frank Griesinger, Oldenburg<br>Prof. Dr. Norber Niederle, Leverkusen<br>Dr. Rolf Fimmers, Bonn

Lung Cancer Group Cologne (LCGC)

Chairman: Prof. Dr. J. Wolf
LCGC Coordination Center: Marion Schwartzkopff
Department I of Internal Medicine, University Clinic Cologne
Joseph-Stelzmann Str. 9
50931 Köln
Tel: 0221 / 478-3410
Fax: 0221 / 478-6733
marion.schwartzkopff@uk-koeln.de

0.2 Declaration by the trial investigators

In January 1997, the international conference on harmonization issued the „Note for Guidance on Good Clinical Practice“ (ICH-GCP). The studies of the LCGC conform, regarding planning, execution and analysis, to the basic principles of GCP, adapted to the requirements and conditions applying to quality assurance protocols. The studies adhere to the declaration of Helsinki.

The principal investigator and co-investigators undertake to adhere to the declaration of Helsinki and the basic principles of GCP and undertake to publish the results of the trials following completion of data analyses.

Signature

Köln, 01.02.2007

Prof. Dr. Jürgen Wolf

Dr. Thomas Zander

Dr. Karin Töpelt

Dr. Lucia Nogova

0.3 Protocol synopsis

Study Title	A Phase II Clinical Trial to Evaluate the Accuracy of FDG-/FLT-PET for Early Prediction of Non-Progression in Patients with Advanced Non Small Cell Lung Cancer (NSCLC) treated with Erlotinib and to Associate PET Findings with Molecular Markers
Short Title	ERLOPET - Erlotinib and sequential PET
Protocol Version	V3.00, 19.12.2007
Study Drug	Erlotinib
Study Design	Clinical phase II study (therapeutic exploratory), single-arm, single-center, open-label
Primary Objective	To evaluate the accuracy of FDG-/FLT-PET analyses for early prediction non-progression in patients with NSCLC treated with Erlotinib
Secondary Objectives	1. To identify PET characteristics for progressive disease following erlotinib treatment<br>2. To identify PET characteristics for response to erlotinib<br>3. To identify PET characteristics for stable disease following erlotinib treatment<br>4. To confirm the statistical association of EGFR mutational status and clinical response in NSCLC patients treated with erlotinib<br>5. To confirm the statistical association of KRAS mutational status and clinical resistance to treatment of NSCLC patients with erlotinib<br>6. To determine the discriminatory capability of FDG-/FLT-PET regarding EGFR and KRAS mutational status in NSCLC patients<br>7. To compare prognostic value of FDG-PET, FLT-PET and EGFR mutational status regarding clinical response in NSCLC patients treated with erlotinib<br>8. To identify EGFR and KRAS sequence characteristics for clinical response and stable disease following erlotinib treatment<br>9. To assess safety of Erlotinib<br>10. To determine response rates<br>11. To determine rates of stable diseases<br>12. To determine one-year FFTF<br>13. To determine one-year OS<br>14. To determine median overall survival time
Investigators	Principal investigator: Prof. Dr. Jürgen Wolf<br>Co-investigators: Dr. Thomas Zander, Dr. Lucia Nogová, Dr. Karin Töpelt, Dr. Roman Thomas, Matthias Scheffler, Dr. Andreas Draube, Dr. Martin Weihrauch
PET	PD Dr. Markus Dietlein, PD Dr. Matthias Schmidt, Dr. Carsten Kobe, Juliane Bachmann

Radiology	Dr. Christopher Bangard, Prof. Dr. Barbara Krug, Dr. Gesa Eyl
Pathology	PD Dr. Claudia Wickenhauser
Statistical Analysis	PD Dr. Martin Hellmich
Study Center(s)	Department I of Internal Medicine, University Clinic Cologne, Joseph-Stelzmann Str. 9, 50931 Cologne, Germany
Number of Patients	40 patients
Treatment Schedule(s)	• week 1-6 (day 1-42): erlotinib p.o., 150 mg once daily<br><br>After the 6 week treatment period with erlotinib it is recommended to continue erlotinib treatment in patients with clinical benefit (CR,PR,SD) until progression and to start standard chemotherapy in patients with progressive disease and a good overall performance state.
Duration of Treatment	Erlotinib treatment for 6 weeks followed by response assessment and continuation of erlotinib treatment in patients with clinical response and stable disease.
Study Period	12 months
Recruitment Start Date	Oct 1, 2007
Recruitment Finish Date	Jan 1, 2009
Follow-up Period End Date	Aug 15, 2010
Inclusion Criteria	• Written informed consent<br>• ≥ 18 years of age<br>• Untreated non-small cell lung cancer stage IIIB/IV<br>• Life expectancy > 3 months<br>• Performance status ECOG 0-2
Exclusion Criteria	• Concurrent systemic immune therapy, chemotherapy or therapy with any anticancer drug not indicated in the study protocol<br>• Any investigational agent(s) within 4 weeks prior to study entry<br>• Previous administration of any EGFR-targeted therapy (antibodies, small molecules and others)<br>• Any medical, mental or psychological condition which in the opinion of the investigator would not permit the patient to complete the study or understand the patient information
Insurance	Gerling – Universitätsklinikum Köln
Monitoring/Datamanagement	ZKS Köln
Statistics	ROC analysis using SUV of FDG-/FLT-PET. Statistical association of molecular status and clinical response (Fisher´s

	exact test)

0.4 Flowsheet

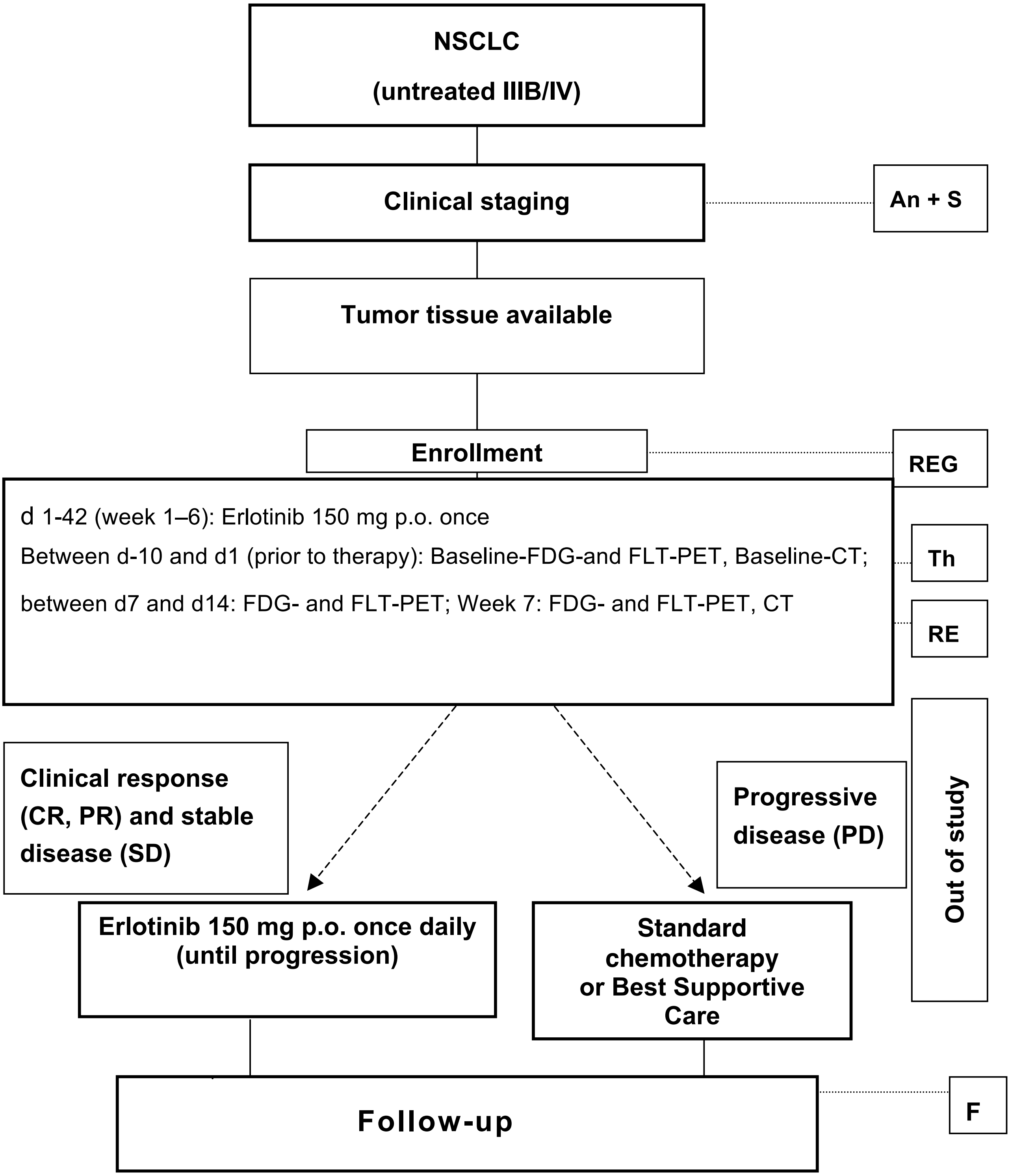

Schedule of CT, FDG-PET and FLT-PET imaging

**Time point**	**Imaging of involved areas**
Between d-10 and d1 (prior to therapy)	FDG-PET, FLT-PET, CT
Day 1	Start of therapy with erlotinib
Between d7 and d14	FDG-PET and FLT-PET
Week 7	FDG-PET, FDG-PET, CT

1 STUDY DESIGN: RATIONALE

1.1 Systemic treatment options in NSCLC

Non small cell lung cancer (NSCLC) is the leading cause of cancer death in the western world (Jemal et al., 2004). While only a minority of patients with early stage disease is cured by radical surgery, most patients present with locally advanced or metastatic disease (stage IIIB, stage IV). None of these patients will be cured and the overwhelming majority dies within the first year after diagnosis. Chemotherapy has been shown to increase survival and to improve quality of life in these patients. However, the increase in median survival time is marginal, i.e. in the range from 2 to 4 months corresponding to an increase in the 1 year survival rate in the range from 10 – 20% (Spira and Ettinger, 2004). In patients with a good performance state the administration of a standard two drug chemotherapy regimen, not necessarily being platinum-based (D'Addario et al., 2005), is considered standard treatment. No significant differences could be observed between several of such standard regimens (Schiller et al., 2002). Similar effects, however, have also been demonstrated in older patients with monochemotherapy alone. Recently, in a large randomized trial the addition of the monoclonal anti-VEGF antibody bevacizumab to paclitaxel/carboplatin first line chemotherapy has been shown to cause an overall survival benefit of two months in non squamous cell NSCLC (Sandler, 2005). Nearly all patients suffer from relapse of the disease with a median survival time of only a few months following diagnosis of relapse. Several chemotherapeutic agents, among others docetaxel, gemcitabine, vinorelbine, exert therapeutic activity in these patients and even a moderate effect on survival compared to best supportive care could be shown for docetaxel (Shepherd et al., 2000). In addition, the new multitargeted antifolat pemetrexed exerts a therapeutic activity comparable to docetaxel, however, with a better tolerability (Hanna et al., 2004).

1.2 EGFR-targeted therapy in NSCLC

The disappointing results of chemotherapy stimulated an intense search for new therapeutics in NSCLC. The knowledge of the particular changes in molecular signaling pathways involved in malignant transformation and tumor progression has led to the generation of novel drugs (“biologicals”) that specifically target at signaling intermediates of oncogenic pathways (e.g. small-molecule inhibitors of tyrosine kinases) or growth factor receptors on the cell surface (e.g. monoclonal antibodies). Several of these drugs have already been established in other solid tumors. Well-known examples are the monoclonal antibody (Mab) trastuzumab targeting HER2 in HER2-expressing breast cancer (Cobleigh et al., 1999) and the tyrosine kinase inhibitior (TKI) imatinib mesylate in chronic myelogenous leukaemia (Druker et al., 2001). In NSCLC the epidermal growth factor receptor (EGFR) represents an attractive target for such innovative drugs. The EGFR (HER1) is a member of the HER growth factor receptor family of tyrosine kinases. Activation of this receptor tyrosine kinase activates an intracellular signal transduction cascade with effects on cell growth, survival, angiogenesis and differentiation. The EGFR pathway is thus regarded as pivotal for the regulation of malignant growth in several cancers, among them NSCLC (Yarden, 2001). Three EGFR-targeted “biologicals” have already shown therapeutic benefit in NSCLC, i.e. one monoclonal antibody and two small-molecule tyrosine kinase inhibitors. The Mab cetuximab (Erbitux^{R}), which recently has received FDA approval for treatment of relapsed

colorectal cancer, has been shown to improve response rates in NSCLC when added to platinum-based chemotherapy (Gatzemeier, 2003). The two oral TKI gefitinib (Iressa^R) and erlotinib (Tarceva^R) induce clinical responses in about 10% of relapsed NSCLC patients and stable disease in about 40% (Fukuoka et al., 2003; Kris et al., 2003; Shepherd et al., 2005). Chemotherapy-typical toxicity is avoided with acne-like skin lesions and diarrhoe representing the most prominent side effects. Recently, in a phase III multicenter trial of the National Cancer Institute of Canada a survival benefit was reported for relapsed NSCLC patients treated with erlotinib with a median survival time of 6.7 months (placebo: 4.7 months; (Shepherd et al., 2005). By comparison, despite impressive clinical responses in a subset of patients, no overall survival benefit in patients with relapsed NSCLC could be shown for gefitinib compared to placebo in a recently published randomized trial (Barge, 2004). Gefitinib had already received approval for the treatment of NSCLC patients after two chemotherapeutic treatment regimens in the USA, Japan, Australia and Switzerland due to the impressive clinical responses. But, until the final analysis of the data of the ISEL trial and further ongoing trials the administration of gefitinib is no more recommended (Barge, 2004). In the US and in the EU, erlotinib is approved for therapy of relapsed NSCLC after failure of at least one chemotherapy. Recently, first-line treatment of NSCLC patients in advanced stage (IIIB/IV) with erlotinib was evaluated in a phase II study and impressive clinical responses were reported. The non-progression rate was 55%. Best response was: 1 CR, 12 PR, 16 SD, 17 PD, 7 NE in this cohort (Giaccone, 2005). Furthermore, Jackman and colleagues performed a study in elderly patients with erlotinib monotherapy as a first line treatment. Efficacy was demonstrated with median 1 year survival rate of 46% and a median overall survival of 10.9 months comparable to results expected in the firstline treatment with combined platinum based chemotherapy (Jackman et al., 2007) (Schiller et al., 2002). In a German study using gefitinib monotherapy in the first line setting the progression free survival was described as 7 weeks (Reck et al., 2006). Further studies to test erlotinib in the line setting progression free survival are ongoing.

1.3 Correlation of EGFR/ KRAS mutations and EGFR immunohistochemistry to clinical benefit

Mutations in the EGFR (frequency 2-25%) correlate with response (partial response, complete response) to EGFR-TKI (Lynch et al., 2004; Paez et al., 2004) in patients with NSCLC. These mutations were found within the kinase-domain-encoding region of the EGFR gene in tumor DNA of erlotinib and gefitinib responders, but not in that of non-responders. These results suggest that the kinase domain mutations may lead to enhanced binding of erlotinib and gefitinib in the ATP-binding pocket of the kinase domain, and thus, to enhanced inhibition of the tyrosine kinase and, ultimately, to enhanced tumor control. The kinase domain of the EGFR gene is encoded by seven exons and can reliably be detected in the tumor tissue only when the tumor-cell population represents more than ~70% of the affected tissue.

Protein expression of EGFR assessed by immunohistochemistry has been demonstrated to be predictive for clinical response to treatment with either gefitinib or erlotinib (Cappuzzo et al., 2005; Shepherd et al., 2005).

Interestingly, there is a group of patients without mutations within the EGFR also responding to inhibition with EGFR-TKI (Pao et al., 2004). In addition, a substantial proportion of patients

treated with EGFR-TKI experience a clinical benefit by achieving a stable disease state which is not correlated to EGFR mutations. Moreover, in the randomized trial (Tarceva vs. placebo) the survival benefit for patients with Tarceva was not correlated to EGFR mutational status (Shepherd et al., 2005; Tsao, 2005). Thus, although EGFR mutations are highly correlated to response to EGFR-TKI, they do not predict clinical benefit and survival.

Mutations within KRAS have a negative prognostic impact on clinical outcome in NSCLC (Rodenhuis and Slebos, 1990). Activating mutations within the KRAS gene are found in 15-50% of lung adenocarcinoma and are mostly localized within exon 2 (Rodenhuis et al., 1988; Suzuki et al., 1990). It has recently been shown that mutations within KRAS confer resistance to the treatment with erlotinib and gefitinib and are mutually exclusive with EGFR mutations (Pao et al., 2005). Based on their low frequency of about 15% and a 50-60% progressive disease rate it is well conceivable that KRAS mutations do not represent the only determinant of resistance to erlotinib respectively gefitinib treatment.

Taken together, sequence analyses of EGFR or KRAS for pre-treatment prediction of clinical response will likely miss a subset of responders that might have experienced substantial clinical benefit from EGFR-targeted therapy (i.e. non-mutated responders). Further the mutational status of those patients with stable disease is not yet clear. Moreover, these analyses are presumed to be only rarely applicable in clinical routine diagnostic procedures. So far, no assays are available that identify those patients definitely not profiting from EGFR-TKI treatment, i.e patients with progressive disease under treatment.

1.4 FDG- and FLT-PET for assessment of response to treatment

The addition of FDG (fluoro-deoxy-glucose)-PET to conventional pre-treatment workup has now become part of the guidelines of the American Society of Clinical Oncology (ASCO) for the diagnostics of patients with lung cancer (Pfister et al., 2004). This is due to an increase in specificity and sensitivity as compared to conventional CT scans (Pieterman et al., 2000; van Tinteren et al., 2002). In addition, substantial progress in measurement of tumor cell proliferation by PET has been made by synthesis of FLT (fluoro-L-thymidine, (Shields et al., 1998). FLT is trapped in tissue after phosphorylation by thymidine kinase 1 (TK1), resulting in high accumulation in tumors compared to non malignant tissue. In NSCLC, a high correlation was found between standardized uptake value (SUV) measures of FLT uptake and Ki-67 scores, an established marker of tumor cell proliferation (Buck et al., 2003; Buck et al., 2002). In these studies, the specificity was 100% and the sensitivity was ~85%. These findings suggest FLT-PET to become an ideal complementary technology to FDG-PET due to its higher specificity. Here, we hypothesize that a decrease in tumor cell proliferation as an early event during the response to erlotinib may be visualized by FDG- and/or FLT-PET and, vice versa, absence of this response may predict early progress. This hypothesis is mainly built on the following findings: Tumor cell proliferation was rapidly reduced by erlotinib in an in-vitro model of lung adenocarcinoma (Pao et al., 2004). Since at least FLT-PET was shown to directly correlate to tumor cell proliferation (Buck et al., 2003; Buck et al., 2002), we assume that FDG-/FLT-PET will allow to early determine response status of a patient under erlotinib treatment. Further clinical and experimental data support our hypothesis: In several early clinical studies it has been demonstrated for different malignancies such as breast cancer (Rousseau, 2005), soft tissue sarcoma (Schuetze, 2005), glioblastoma (Charnley NG, 2005), rectum cancer (Di Fabio, 2005) and lung cancer (Griesinger, 2005; Schmuecking,

2005) that clinical response to chemotherapy can be detected by FDG-PET after 4-8 weeks. Sensitivity for response prediction outperformed conventional imaging in these studies. In squamous cell cancer of head and neck it has been demonstrated that FDG-PET after completion of treatment with an anti-EGFR antibody depicts response consistently (L Bastholt, 2005). Furthermore, not only response to treatment, but also clinical progression can be detected reliably by FDG-PET in mesothelioma (Kimura, 2005). In an in vivo model of fibrosarcoma Leyton and colleagues detected a decrease in FLT uptake as early as 24h after start of chemotherapy with cisplatin. In this model FLT outperformed FDG as tracer (Barthel et al., 2003; Leyton et al., 2005). Similar data were obtained in an allograft mouse model of a squamous cell tumor as well as an cervical adenocarcinoma model (Sugiyama, 2004). Moreover, in recent studies FDG-PET was shown to reliably predict early response of patients with gastrointestinal stromal tumors (GIST) to the small molecule tyrosine kinase inhibitor imatinib mesylate or SU11248 (Jager et al., 2004; Stroobants et al., 2003; van den Abbeele, 2005) on day 8. Also response of GIST to treatment with the mTOR inhibitor AP23573 can be detected by FDG-PET already on day 5 of treatment (Sankhala, 2005).

2 AIM OF TRIAL

Based on the survival benefit demonstrated in patients with relapsed NSCLC (Shepherd et al., 2005) as well as on its favourable toxicity profile compared to chemotherapy the EGFR-TKI erlotinib represents an attractive candidate to be included also in protocols for the first line treatment of NSCLC. Accordingly, preliminary data from phase II trials show promising response rates for erlotinib treatment in the first line setting (Giaccone, 2005). In parallel, in the near future further targeted drugs will be available for NSCLC treatment. For instance, the addition of the anti-VEGF monoclonal antibody bevacizumab has recently shown to prolong survival when added to standard chemotherapy as first line treatment of NSCLC. Thus, in order to optimize the choice and the sequence of new drugs in NSCLC treatment, an early identification of patients with no clinical benefit, i.e. patients with progressive disease under treatment, is desirable. In the ERLOPET trial, we aim at establishing PET analysis as a tool for early identification of NSCLC patients with no clinical benefit from monotherapy with erlotinib as first line treatment. A pre-treatment computed tomography (CT) scan will be performed for evaluation of the patients´ individual tumor burden. FDG- and FLT-PET scans will be performed between d -10 and d1 (but prior to therapy) for identification of pre-treatment PET status. Between d7 and d14, FDG- and FLT-PET scans will be performed for the early identification of erlotinib non responders. The imaging procedures will be performed at different days, i. e. one procedure per day. After six weeks of treatment, final response evaluation will be performed using CT, FDG- and FLT-PET scans. These results will be correlated to the mutational status of the EGFR - and the KRAS gene and the expression of EGFR in pretreatment samples.

3 TRIAL PLAN

3.1 Trial design, primary and secondary objectives

Design

Phase II study (therapeutic exploratory), single center, single arm, open-label

Primary Objective

To evaluate the accuracy of FDG-/FLT-PET analyses for early prediction of erlotinib non-response in patients with stage IIIB/IV NSCLC (measured by ROC analysis of the SUV) treated with erlotinib

Secondary Objectives

1. To identify PET characteristics for progressive disease following erlotinib treatment (changes in SUV during treatment)
2. To identify PET characteristics for response to erlotinib (changes in SUV during treatment)
3. To identify PET characteristics for stable disease following erlotinib treatment (changes in SUV during treatment)
4. To confirm the statistical association of EGFR mutational status and clinical response in NSCLC patients treated with erlotinib
5. To confirm the statistical association of KRAS mutational status and clinical resistance to treatment of NSCLC patients with erlotinib
6. To determine the discriminatory capability of FDG-/FLT- PET regarding EGFR and KRAS mutational status in NSCLC patients
7. To compare prognostic value of FDG-PET, FLT-PET and EGFR mutational status regarding clinical response in NSCLC patients treated with erlotinib
8. To identify EGFR and KRAS sequence characteristics for clinical response and stable disease following erlotinib treatment
9. To assess safety of Erlotinib
10. To determine response rates
11. To determine rates of stable diseases
12. To determine one-year FFTF
13. To determine one-year OS
14. To determine median overall survival time

3.2 Recruitment

It is planned to recruit a maximum of 40 patients over a 15 months period.

Patients will be recruited after screening only if all inclusion criteria are met, no exclusion criteria are met and written informed consent has been obtained. No difference in recruitment concerning gender will be applied. According to the distribution of NSCLC more male than female study participants are expected.

3.3 Duration of the study

The expected duration from beginning of recruitment to final data analysis will be 33 months (15 months recruitment, 15 months treatment (6 weeks for each patient, further treatment after 6 weeks is not part of the study), 18 months follow-up).

First patient in: 1.10.07; Last patient out: 15.2.2009, Closure of the database: 15.8.2010

3.4 Termination of protocol treatment

The patient can discontinue trial participation at his/her own wish at any time. Protocol treatment can be terminated based upon the decision of the treating physician, due to unacceptable toxicity, progression, serious intercurrent disease or pregnancy. In any case, following a formal or an actual termination, the patient's follow up should be documented.

3.5 Early termination of trial

The trial can be terminated early by the principal investigator in consultation with the co-investigators, if the formal termination criterion is reached or if patients' safety appears to be at risk. In case of termination of the trial the review board will be informed.

4 ENROLLMENT

4.1 Inclusion criteria

1. First diagnosis of histologically proven non-small cell lung cancer stage IIIB/IV
2. Tumor tissue available and suitable for EGFR gene resequencing
3. At least one measurable lesion according to RECIST
4. No prior standard chemotherapy, immunotherapy or small-molecules therapy
5. ≥ 18 years of age
6. ECOG performance status 0-2
7. Patient's own, written informed consent to participate in the trial
8. TSH, FT3 and FT4 serum levels compatible with contrast enhanced CT

   (no signs of hyperthyroidism)
9. Renal laboratory parameters compatible with contrast enhanced CT

   (serum creatinine ≤ 1,7 mg/dl)
10. No decompensated liver failure
11. No decompensated heart failure
12. No clinical or radiological sign of interstitial lung disease, no interstitial lung disease in the past
13. Life expectancy > 3 months
14. In case of female patients with childbearing potential:
    - negative pregnancy test
    - effective method of contraception

4.2 Exclusion criteria

1. Lack of appropriate biopsy specimen for EGFR gene re-sequencing
2. Previous systemic treatment of NSCLC (chemotherapy, immune therapy, therapy with small molecules and others)
3. Concurrent systemic immune therapy, chemotherapy, or therapy with any anticancer drug not indicated in the study protocol
4. Solitary metastases accessible to surgical resection
5. Bone metastases which have to be treated by radiotherapy (e.g. impending fracture), if no additional metastases outside the radiation field are present
6. Tumor masses with acute risk of life-threatening complications (e.g. compression of large arteries or trachea) which require emergency treatment (e.g. radiotherapy)
7. Malignant disease other than NSCLC within the last 5 years

   (exceptions: basalioma, carcinoma in situ of the cervix uteri, completely resected melanoma pT1N0M0 (Clark level II))
8. Infection with HIV, Hepatitis B, Hepatitis C
9. Patient's lack of accountability, inability to appreciate the nature, meaning and consequences of the trial and to formulate his/her own wishes adequately
10. Any medical, mental or psychological condition which in the opinion of the investigator would not permit the patient to complete the study or understand the patient information
11. Any person being in an institution on assignment of the respective authority
12. Simultanous participation in any other clinical trial
13. Pregnancy, lactation
14. Diabetes mellitus
15. Elevated fasting blood glucose levels > 130 mg/dl (exception: reduction of an unexpectedly high blood glucose level to a value ≤ 130 mg/dl using regular insulin)

5 INDIVIDUAL TRIAL PROCEDURE

5.1 Diagnosis and allocation to trial

Pathological diagnosis

Diagnosis of NSCLC will be made/confirmed in the Institute for Pathology, University Clinic Cologne.

Procurement of biopsy material for biological analyses

In case of study inclusion material that is not required for histological diagnosis will be stored under the respective conditions (room temperature for paraffin-embedded tissue, -80° C in a

phone-alarm secured freezer in a local tissue bank of MTBTI in case of frozen or fresh material).

The following persons are responsible for the safe processing and storage of all biological material obtained during the study:

Dr. Thomas Zander
Dr. Karin Töpelt
Dr. Lucia Nogova
Molecular Tumor Biology and Tumor Immunology, Haus 16
Department I of Internal Medicine, University Clinic Cologne
Joseph-Stelzmann Str. 9
50931 Koeln
Telefon: 0221-478-5413 / 4447
Fax: 0221-478-3647

5.2 Pseudonymization

After inclusion into the trial each patient will be given a consecutive number. All documents (CRF) will be marked only with this number. Personal data and anonymous number will be stored separately.

5.3 Mutation analysis

Processing of tissue will be performed according to standard operating procedures (SOPs) established in MTBTI. Pretreatment biopsy material (tumor purity >70%, alternatively: tumor cell enrichment by microdissection) will be analyzed for EGFR mutational status by exon sequencing. A PCR has been established that allows for amplification and direct sequencing of EGFR mutational hotspots in exons 18, 19 and 21 from paraffin-embedded tumor tissue. Further a PCR amplification of KRAS exon 2 was established in the MTBTI (Zander et al. in preparation). In control experiments that were carried out on non-selected paraffin-embedded lung cancer specimens from a local tissue bank, PCR efficiency for all target amplicons was 100%. Target sequences will be amplified by single-round PCR. Purified PCR products will be bi-directionally sequenced (sequences controlled for bi-allelic target amplification; quality threshold: Phred score >30). Electropherograms will be manually and computationally (software: DNAsis) compared to germline sequences. In those tumors where tumor cell purity is estimated to be <70%, tumor cells will be enriched by laser-assisted microdissection (PALM system, available through a close collaboration with the Institute of Pathology, University of Cologne). All technology is established in the MTBTI laboratories and there is extensive experience with generating and evaluating molecular genetic data.

5.4 EGFR immunohistochemistry

A positive correlation between EGFR positivity in immunohistochemistry and response to therapy has been demonstrated in relapsed NSCLC patients treated with erlotinib in the BR.21 phase III trial (Shepherd et al., 2005). Therefore, EGFR expression in primary paraffin embedded tumor tissue will be analyzed. EGFR staining will be performed in the Institute of

Pathology of the University Hospital Cologne following standardized procedures established for clinical application.

5.5 Pre-Study Screening (staging examination)

Within a maximum of 14 days prior to registration, all patients will be screened by the investigator. Each patient must provide a written informed consent to the trial procedures. The patient must be informed about the study verbally and by the patient information by the principal investigator or one of the co-investigators, before informed consent is obtained. After obtaining written informed consent, the patient will be screened for eligibility. The following will be assessed:

- Patient demography
- Clinical examination including physical examination:
  - height, weight, vital signs, ECOG performance status
- Assessment of the neurological status
- Existing signs and symptoms
- Medical history (including concurrent illnesses) and review of all diagnostic procedures already performed for first diagnosis and staging of NSCLC
- Previous anti-cancer therapies and their outcome
- Concomitant medication
- Laboratory assessment including:
  - blood count including differential count
  - serum electrolytes (Na, K, Ca),
  - serum liver function parameters (total bilirubin, ASAT, ALAT, AP, γGT, LDH),
  - serum renal function parameters (creatinine, BUN, uric acid),
  - serum TSH, FT3, FT4
  - total serum protein
  - ESR
  - blood coagulation parameters (Quick, PTT, Fibrinogen),
  - screening for infecton with HIV1/2, hepatitis B, hepatitis C
  - CYFRA 21-1, CEA
  - fasting glucose level
  - pregnancy test for women with childbearing potential
- MRT or CT scan of the skull
- Electrocardiography (ECG)
- Cardiac function (LVEF) by echocardiography
- Pulmonary function (PO2, PCO2, DCO, vital capacity FEV1)
- FDG- and FLT-PET (s. table below)

- CT scans of all involved areas, as indicated in preexisting diagnostic procedures or PET analyses (s. table below).

Table 1. Schedule for CT, FDG-PET and FLT-PET imaging.

Time point	Imaging of involved areas
Between d-10 and d1 (prior to therapy)	FDG-PET, FLT-PET, CT
Day 1	Start of therapy with erlotinib
Between d7 and d14	FDG-PET and FLT-PET
Week 7	FDG-PET, FDG-PET, CT

5.6 PET

Patient preparation

For FDG-PET patients must refrain from ingestion for 12 hours prior to the PET examination; only the ingestion of carbohydrate-free drinks is permitted. The current glucose level should be measured before starting PET. Fasting glucose level over 130 mg/dl or known diabetes mellitus are exclusion criteria. Optionally regular insulin (e.g. 2 units) may be administered if blood sugars are unexpectedly high, provided that monitoring and emergency care are assured. For FLT-PET no special preparation is necessary.

Administration of tracers, scanning and data evaluation

18F-FDG and 18F-FLT will be synthesized at the Max-Planck-Institute for Neurological Research at the University of Cologne following standard procedures. A dose of 370 MBq FDG and 300 MBq FLT will be injected intravenously for whole-body examination by PET. Scanning will commence ~45-60 minutes after injection of tracers. PET instruments with full-ring detectors will be applied. Emission data will be corrected for attenuation, scatter and random and will be reconstructed to plains of 3.125 mm width and 128x128 voxels. For quantification of tracer uptake, standard methods, such as standardized uptake values (SUV) will be applied.

Applied dose in each PET analysis:

Total activity for the respective RP	ED due to application of the RP	ED due to application of ALL the RP's	ED of all TM's	Allover ED
Max. 370 MBq FDG<br>Day -4	7,0 mSv	7 mSv	0,1 mS	**7,1 mSv**
Max. 300 MBq FLT<br>Day -3	9,9 mSv (f)<br>8,4 mSv (m)	16,9 mSv (f)<br>15,4 mSv (m)	0,1 mSv	**17,0 mSv (f)**<br>**15,5 mSv (m)**
Max. 370 MBq FDG<br>Day +8	7,0 mSv	23,9 mSv (f)<br>22,4 mS (m)	0,1 mS	**24,0 mSv**<br>**22,5 mSv**
Max. 300 MBq FLT<br>Day +9	9,9 mSv (f)<br>8,4 mSv (f)	33,8 mSv (f)<br>30,8 mSv (m)	0,1 mSv	**33,9 mSv**<br>**30,9 mSv**
Max. 370 MBq FDG<br>Week 7	7,0 mSv	40,8 mSv (f)<br>37,8 mSv (m)	0,1 mS	**40,9 mSv**<br>**37,9 mSv**
Max. 300 MBq FLT<br>Week 7	9,9 (f) mSv<br>8,4 (m) mSv	50,7 mSv (f)<br>46,2 mSv (m)	0,1 mSv	**50,8 mSv (f)**<br>**46,3 mSv (m)**

The used dose is the minimal dose to obtain high quality data suitable for further analysis

5.7 Patient briefing and termination of protocol treatment

After completion of staging, the patient is informed about the trial, including the following aspects: title and aim of the trial, nature of the treatment, side effects, reason for recruitment, passing on of data and material samples, insurance, the ethics committee vote and the patient's freedom to decide. The patient receives an information brochure and an informed consent form.

The signed original consent form and the record of the briefing remain in the patient's files. The patient will receive a copy of each document.

The patient can make a free decision on participation in the trial at any time. If trial participation is refused, the treatment can be freely chosen in accordance with the treating physician.

Termination of protocol treatment can occur at the patient's wish or if the treating physician decides to terminate it due to unacceptable toxicity, serious concurrent disease or pregnancy. Documentation after termination will be performed

Informed consent

The investigator assumes the responsibility of obtaining written informed consent for each patient or the patient's legally authorized representative before any trial-specific procedures are performed and before any trial drug is administered.

Patients meeting the criteria set forth in the protocol will be offered the opportunity to participate in the trial. To avoid introduction of bias, the investigator must exercise no selectivity with regard to offering eligible patients the opportunity to participate in the trial. Patients or parents/legal guardians of all candidate patients will receive a comprehensive explanation of the proposed treatment, including the nature of the therapy, alternative therapies available, any known previously experienced adverse reactions, the investigational status of the trial drug, and other factors that are part of obtaining a proper informed consent. Patients will be given the opportunity to ask questions concerning the trial, and adequate time to consider their decision to or not to participate.

Informed consent will be documented by the use of a written consent form that includes all the elements required by regulations and ICH guidelines. The form is to be signed and dated by the patient or patient's legally authorized representative and by the person who administers the consent process. A copy of the signed form will be given to the person who signed it, the original signed consent form will be filed with the patient’s medical records. The date and time of the informed consent must be recorded in the source documents.

If an amendment to the protocol changes the patient participation schedule in scope or activity, or increases the potential risk to the patient, the informed consent document must be revised, submitted to the EC for review and approved by the EC before use. The revised informed consent document must be used to obtain re-consent from any patients currently enrolled in the trial if the patient is affected by the amendment, and must be used to document consent from any new patients enrolled after the approval date of the amendment.

Patient privacy

The investigator affirms and upholds the principle of the patient's right to privacy. The investigators shall comply with applicable privacy laws.

Confidentiality

The written informed consent form will explain that the trial data will be stored in a computer database, maintaining confidentiality in accordance with local data legislation and the EU directive 95/46/EG. Subjects in this database will be identified by patient identifier only. The patient information will also explain that for data verification purposes, authorized representatives of the sponsor, a regulatory authority, an ethics committee, or an institutional review board may require direct access to parts of the medical records relevant to the trial, including subjects’ medical history.

Especially, anonymity of the patients will be guaranteed when presenting the data at scientific meetings or publishing them in scientific journals.

5.8 Enrollment into the trial

Reporting and recruitment

If after completion of staging the patient is qualified for the trial and he/she gives written informed consent, recruitment will be performed by LCGC in Köln. For recruitment, the following is required:

- all entry criteria satisfied;
- no exclusion criterion satisfied;
- staging procedures completed according to protocol.

Patients should be asked to agree to be contacted by the trials coordination center.

This agreement and the patient's address are to be recorded on the REG form.

Patients who wish to avoid sending their personal data to the trials coordination center may be enrolled anonymously under a pseudonym code. Prerequisite for anonymous enrollment is, however, commitment to follow-up and documentation.

5.9 Treatment

Erlotinib (Tarceva®)

The oral drug erlotinib (OSI-774, OSI pharmaceuticals; Tarceva[R], Roche) is a small-molecule EGFR tyrosine kinase inhibitor.

Three phase-II single agent studies with erlotinib at 150 mg daily dose were performed (I) in patients with chemotherapy resistant, HER1/EGFR-expressing NSCLC of all histological sub-entities (Perez-Soler et al., 2004), (II) in patients with bronchoalveolar carcinoma previously untreated or treated with chemotherapy (Patel, 2003) and (III) as first-line therapy in elderly patients with NSCLC of all histological subentities (Johnson, 2004). The drug was well tolerated, drug-related cutaneous rash and diarrhea were observed in approximately two thirds of patients. The response rates were 12.3%, 25% and 13.3%, respectively. The median survival was 8.4 months, and the 1-year survival rate was 40% (Perez-Soler, 2004). A recently presented study with erlotinib at 150 mg as single agent in first line treatment of IIIB/IV NSCLC patients showed a non-progression rate of 55% after 6 weeks of treatment (Giaccone, 2005).

Based on the results of the single agent phase II studies, phase III studies in combination with chemotherapy were performed. In two large phase III studies (TALENT, TRIBUTE) analyzing treatment of erlotinib in combination with chemotherapy, in more than 2000 patients no benefit for the addition of erlotinib could be shown (Gatzemeier, 2004; Herbst, 2004).

Recently, the randomized, double blind, placebo controlled study performed by the National Cancer Institute of Canada evaluated 731 patients with locally advanced or metastatic (stage IIIb or IV) NSCLC after failure of one or more chemotherapy regimens. The proportion of patients who experienced an objective response in the erlotinib monotherapy group was higher than in the placebo group (8.9% versus 0.9%, $p<0.001$). Treatment with erlotinib was associated with significantly longer overall survival than placebo (median survival 6.7 months versus 4.7 months respectively) (Shepherd et al., 2005).

These data led to FDA approval in the US in November 2004. With the U.S label, Tarceva® (erlotinib) is indicated for all NSCLC patients after failure of at least one prior chemotherapy regimen. In 2005 Tarceva® has been approved in Switzerland and in the EU for the same indication.

Storage of Tarceva:

Tarceva will be provided by Roche and stored at room temperature in a temperature controlled, locked locker in the outpatient department of the department of internal medicine I. Unlabeled Tarceva® as commercially available will be provided by Roche.

Administration of therapy

Day 1-42 (week 1-6): Erlotinib (Tarceva®) 150 mg p.o. **once** daily,

It is recommended to administer Tarceva® 1 hour after meals. In addition, patients should avoid food ingestion for at least 2 hours after Tarceva®. For the same reason the administration of proton pump blockers is not recommended during therapy.

It is recommended to perform the follwing blood tests routinely once per week during erlotinib (Tarceva®) therapy:

EDTA blood: blood cell count

serum: creatinin, uric acid, bilirubin, gamma-GT, GOT, GPT

Side effects

Reported erlotinib (Tarceva®) adverse reactions

Clinically significant erlotinib (Tarceva®) adverse reactions:

1. Grade 3 to 4 **rash and diarrhea** occurred in 9% and 6% patients, respectively. The median time to onset of rash in a phase III clinical trial was 8 days and to onset of diarrhea 12 days (Shepherd et al., 2005).
2. **Liver function abnormality** - grade 2 (>2.5 – 5.0 x ULN) including elevated ALT, AST and bilirubin occurred in 4% of the patients treated with Tarceva and <1% of placebo treated patients in a phase-III study. Grade 3 elevations were not observed in Tarceva® treated patients. During treatment with erlotinib, the patients should be monitored by liver function tests at least once every 1 to 2 months (Shepherd et al., 2005).
3. **Interstitial pneumonia** (interstitial lung disease – ILD) has been observed in a phase III study in NSCLC patients receiving erlotinib at an overall incidence of about 0.8 percent, which was not different to the placebo group. Among patients who developed ILD, symptoms began within 5 days to more than 9 months after erlotinib therapy initiation (median 47 days). The overall incidence of ILD in Tarceva® treated patients was reported to be approximately 0.6%. In the event of acute onset or worsening of pulmonary symptoms (dyspnea, cough, fever), erlotinib therapy should

be interrupted and a prompt diagnostics of these symptoms should start. If interstitial lung disease is confirmed, erlotinib should be discontinued and the patient should be treated appropriately (Shepherd et al., 2005).

Adverse reactions occurring in Tarceva®-treated patients (Shepherd et al., 2005)

**NCI CTC Grade**	**Any Grade (%)**	**Grade 3 (%)**	**Grade 4 (%)**
Rash	75	8	<1
Diarrhea	54	6	<1
Anorexia	52	8	1
Fatigue	52	14	4
Dyspnea	41	17	11
Cough	33	4	0
Nausea	33	3	0
Infection	24	4	0
Vomiting	23	2	<1
Stomatitis	17	<1	0
Pruritus	13	<1	0
Dry skin	12	0	0
Conjunctivitis	12	<1	0
Keratoconjunctivits	12	0	0
Abdominal pain	11	2	<1

Precautions

1. Potent inducers of CYP3A4 may reduce the efficacy of erlotinib whereas potent inhibitors of CYP3A4 may lead to increased toxicity. Concomitant treatment with these types of agents should be avoided.
2. Current smokers should be advised to stop smoking, as plasma concentrations could be reduced otherwise.
3. Cases of interstitial lung disease (ILD), including fatalities, have been reported uncommonly in patients receiving Tarceva for treatment of non-small cell lung cancer (NSCLC) or other advanced solid tumours.
4. If a **patient develops diarrhoea**, the patient should be managed appropriately by symptomatic treatment, dose reduction or temporary suspension of the treatment with

erlotinib depending on the condition of the patient (see chapter 6.5 Treatment postponement, dose reductions and interruption).

5. In patients with elevated liver enzymes prior treatement (total bilirubin, ASAT, ALAT 2 x ULN) liver enzymes will be monitored twice weekly.

Precautions for co-administration with erlotinib (Tarceva®)

Tarceva® should be **administered with care** when co-administered with following drugs:

Drug	Effects
CYP3A4 inducers:<br>**phenytoin, carbamazepine, rifampicin, barbiturates,** etc.	Blood **erlotinib concentration may be decreased**, which may lead to decreased effects of the drug.
CYP3A4 inhibitors:<br>azole **antifungal agent** (i.e. itraconazole), **macrolide** (i.e.erythromycin), **ritonavir**, **indinavir, diltiazem and verapamil**, etc.	Blood **erlotinib concentrations may be increased**, which may lead to increased incidence and severity of adverse drug reactions. Co-administration with **itraconazole** resulted in an 80% increase in the AUC of erlotinib.
**warfarin, cumarin (Marcumar)**	There were reports on **INR elevations and bleeding events** on co-administration with warfarin. When erlotinib is used concomitantly with warfarin, the patient should be monitored for **prothrombine time or INR** at regular intervals.

Restaging procedures during and after erlotinib therapy

Examinations

Since the primary objective of the proposed study is to evaluate the accuracy of FDG-/FLT-PET analysis for early prediction of erlotinib non-progression in patients with NSCLC, the FDG- and FLT-PET examinations will be performed in exactly defined periods before, during and after therapy (s. table below).

**Time point**	**Imaging of involved areas**
Between d-10 and d1 (prior to therapy)	FDG-PET, FLT-PET, CT
Day 1	Start of therapy with erlotinib
Between d7 and d14	FDG-PET and FLT-PET
Week 7	FDG-PET, FDG-PET, CT

In week 7 the following clinical and laboratory analyses will be performed:

- Clinical examination including physical examination:
  - height, weight, vital signs, ECOG performance status
- Assessment of the neurological status
- Existing signs and symptoms
- Concomitant medication
- Laboratory assessment including:
  - blood count including differential count
  - serum electrolytes (Na, K, Ca),
  - liver function parameters (total bilirubin, ASAT, ALAT, AP, γGT, LDH),
  - renal function parameters (creatinine, BUN, uric acid),
  - ESR
  - CYFRA 21-1, CEA

These examinations may not include examinations which might be necessary if chemotherapeutic treatment is planned for progressive disease (see 5.7.6).

Therapeutic procedure after 6 weeks of erlotinib therapy

Only patients with documented (clinical examination, CT, PET) tumor control under erlotinib therapy (CR, PR or, at least, SD in the restaging procedures in week 7) will continue erlotinib therapy until progression of disease. In all patients with documented progress after 6 weeks of therapy erlotinib therapy will be stopped. Further treatment in these patients depends on overall performance state and declared intention of the patient. If the patient and the principal investigator or one of the co-investigators agree upon chemotherapeutic treatment, this treatment will be offered to the patient in the Department I of Internal Medicine, University Clinic Cologne.

5.10 Follow-up examinations after end of treatment

Follow-up examinations

A standardized patient visit on month 3, 6, 9 and 12 comprises the following examinations:

- CT scan of the involved area
- Clinical examination including physical examination:
    - height, weight, vital signs, ECOG performance status
- Assessment of the neurological status
- Existing signs and symptoms
- Concomitant medication
- Laboratory assessment including:
    - blood count including differential count
    - serum electrolytes (Na, K, Ca),
    - liver function parameters (total bilirubin, ASAT, ALAT, AP, γGT, LDH),
    - renal function parameters (creatinine, BUN, uric acid),
    - ESR
    - CYFRA 21-1, CEA

6 ADVERSE EVENTS

6.1 Handling of Safety Parameters

Serious Adverse Events

Any clinical adverse event or abnormal laboratory test value that is serious occurring during the course of the study, irrespective of the treatment received by the patient, must be reported to the trial coordinating center within one working day of knowledge (expedited reporting).

The definition and reporting requirements according to German Drug Law, GCP-V and ICH Guideline for Clinical Safety Data Management, Definitions and Standards for Expedited Reporting, Topic E2 will be adhered.

Any serious adverse event that is unequivocally due to disease progression should not be reported as an AE. It will be documented in the regular CRF. Laboratory abnormalities of at least grade 2 NCI-CTCAE v3.0 will be documented as AEs.

The trial coordinating center will forward any clinical adverse event or abnormal laboratory test value that is serious to Roche within one working day.

The contact person is:

Lilli Esterle, Dr. Christian Busch

Abt. Hämatologie/Onkologie

Emil-Barell-Str. 1

79639 Grenzach-Wyhlen

Fax-Nr: 07624-14-2397

Roche will fulfill reporting requirements to authorities and ethics committees. SUSARs will be distributed to ethics committees and centers by Roche.

Treatment and Follow-up of Adverse Events

Adverse events, especially those for which the relationship to test "drug" is not "unrelated", should be followed up until they have returned to baseline status or stabilized. If a clear explanation is established it should be recorded on the CRF.

Follow-up of Abnormal Laboratory Test Values

In the event of unexplained abnormal laboratory test values, the tests should be repeated immediately and followed up until they have returned to the normal range and/or an adequate explanation of the abnormality is found. If a clear explanation is established it should be recorded on the CRF.

Pregnancy

A female subject must be instructed to stop taking the test "drug" and immediately inform the investigator if she becomes pregnant during the study. Pregnancies occurring up to 90 days after the completion of the test "drug" must also be reported to the investigator. The investigator must report all pregnancies within one working day to the sponsor. The investigator should counsel the subject, discuss the risks of continuing with the pregnancy

and the possible effects on the fetus. Monitoring of the patient should continue until conclusion of the pregnancy.

Pregnancy occurring in the partner of a subject participating in the study should also be reported to the investigator and the sponsor. The partner should be counseled and followed as described above.

6.2 Definitions

An adverse event is any untoward medical occurrence in a patient or clinical investigation subject, administered a pharmaceutical product and which does not necessarily have to have a causal relationship with this treatment.

Adverse reactions are all untoward and unintended responses to an investigational medicinal product related to any dose administered.

A serious adverse event or serious adverse reaction is any experience that suggests a significant hazard, contraindication, side effect or precaution. It is any adverse event that at any dose fulfills at least one of the following criteria:

- is fatal (results in death)
  (NOTE: death is an outcome, not an event)
- is life-threatening
  (NOTE: the term "life-threatening" refers to an event in which the patient was at immediate risk of death at the time of the event; it does not refer to an event which could hypothetically have caused a death had it been more severe.)
- required in-patient hospitalization or prolongation of existing hospitalization
- results in persistent or significant disability/incapacity
- is a congenital anomaly/birth defect
- is medically significant or requires intervention to prevent one or other of the outcomes listed above

Medical and scientific judgment should be exercised in deciding whether expedited reporting to the sponsor is appropriate in other situations, such as important medical events that may not be immediately life-threatening or result in death or hospitalization but may jeopardize the patient or may require intervention to prevent one of the outcomes listed in the definitions above. These situations should also usually be considered serious.

Examples of such events are intensive treatment in an emergency room or at home for allergic bronchospasm; blood dyscrasias or convulsions that do not result in hospitalization; or development of drug dependency or drug abuse.

An unexpected adverse event is one, the nature or severity of which is not consistent with the applicable product information.

Causality is initially assessed by the investigator. With respect to report and documentation obligation (regulatory authorities, ethics committees and other investigators) for Serious Adverse Events, causality can be one of 2 possibilities:

- No (unrelated; equals not drug related).

- Yes (remotely, possibly, probably or definitely drug related).

All adverse events not assessed as definitive "not drug related" by either the investigator or Roche will be considered as adverse drug reaction.

A suspected unexpected serious adverse reaction (SUSAR) is a serious adverse reaction, the nature, or severity of which is not consistent with the applicable product information.

It is important that the severity of an adverse event is not confounded with the seriousness of the event. For example, vomiting which persists for many hours may be severe, but is not necessarily a serious adverse event. On the other hand, stroke which results in only a limited degree of disability may be considered a mild stroke, but would be a serious adverse event.

A serious adverse event occurring during the study or which comes to the attention of the investigator within 28 days after stopping the treatment, whether considered treatment-related or not, must be reported. In addition, a serious adverse event that occurs after this time, if considered related to test "drug", should be reported.

Such preliminary reports will be followed by detailed descriptions later which will include copies of hospital case reports, autopsy reports and other documents.

For serious adverse events, the following must be assessed and recorded on the adverse events page of the Case Report Form: intensity, relationship to test substance, action taken, and outcome to date.

Document and report obligation have to be adhered according to the national and international laws and regulations.

6.3 Treatment postponement, dose reduction and termination.

In case of any adverse event, which may be classified by the investigators as an suspected drug reaction and is not controlled by optimal supportive care, or not tolerated due to symptoms, disfigurement, or interference with normal daily activities, Tarceva® dose may be reduced according to the following schedule:

Starting dose: 150 mg

First reduction: 100 mg

Second reduction: 50 mg.

Within further 2 weeks after dose reduction, the adverse event must have been improved at least by one NCI-CTC grade to NCI-CTC grade < 2. Otherwise, a further dose reduction must be performed. The dose interruption may last maximal 2 weeks if clinically indicated and if the toxicity is not controlled by optimal therapy.

Once a dose was reduced due to toxicity, the dose will not be reescalated, except for Tarceva® induced rash. In case of rash, the dose can be reescalated if the rash is < grade 2 NCI-CTC.

In case of skin reaction and diarrhoea the following dose management and therapy is indicated:

NCI – CTC Grade	Dose Modification	Recommended Therapy
**RASH**		
grade 1	None	None
grade 2	None	minocycline p.o., tetracycline topical, clindamycin topical, silver sulfadiazine topical, prednison p.o.
grade 3	dose reduction, re-escalation by rash ≤ grade 2	as above
grade 4	off study	
**DIARRHEA**		
grade 1 (stools < 4)	None	loperamide
grade 2 (4 – 6 stools)	None	loperamide
grade 3 (stools ≥ 7)	Interrupt	interruption until resolution to grade ≤ 1, restart at reduced dose
grade 4 (life threatening sequelae, e.g. haemodynamic collapse)	off study	

Patients requiring a dose interruption for > 2 weeks due to not controlled adverse events of the Tarceva® medication will be taken off study.

In any case of a grade IV toxicity the patient will be withdrawn from the study. Any grade III toxicity will be reported to the principal investigator within 24 h after notice.

6.4 Handling of FDG and FLT safety

No adverse events were described after administration of FLT and FDG up to now. In any case of adverse event, especially allergic reaction, the next dose of FLT or FDG, respectively, will not be administered.

7 STATISTICS

7.1 Statistical formulation of study objectives and design

Primary Objective

To evaluate the accuracy of FDG-/FLT-PET analyses for early prediction of non-progression in patients with NSCLC treated with erlotinib

Secondary objectives

1. To identify PET characteristics for progressive disease following erlotinib treatment
2. To identify PET characteristics for response to erlotinib
3. To identify PET characteristics for stable disease following erlotinib treatment
4. To confirm the statistical association of EGFR mutational status and clinical response in NSCLC patients treated with erlotinib
5. To confirm the statistical association of KRAS mutational status and clinical resistance to treatment of NSCLC patients with erlotinib
6. To determine the discriminatory capability of FDG-/FLT-PET regarding EGFR and KRAS mutational status in NSCLC patients
7. To compare prognostic value of FDG-PET, FLT-PET and EGFR mutational status regarding clinical response in NSCLC patients treated with erlotinib
8. To identify EGFR and KRAS sequence characteristics for clinical response and stable disease following erlotinib treatment
9. To assess safety of erlotinib
10. To determine response rates
11. To determine rates of stable diseases
12. To determine one-year FFTF
13. To determine one-year OS
14. To determine median overall survival time

Endpoint

Primary endpoint

Non-progression to therapy with erlotinib, FLT and FDG standard uptake values

Secondary endpoints

FLT and FDG standard uptake value, EGFR mutational status, KRAS mutational status, EGFR positivity in immunohistochemistry, safety

One year FFTF, one year OS

Definitions and explanations

Response criteria are according to the Response Evaluation Criteria in Solid Tumors (RECIST) (http://www3.cancer.gov/bip/RECIST.htm)

Evaluation of target lesions

* Complete Response (CR):	Disappearance of all target lesions
* Partial Response (PR):	At least a 30% decrease in the sum of the longest diameter (LD) of target lesions, taking as reference the baseline sum LD
* Progressive Disease (PD):	At least a 20% increase in the sum of the LD of target lesions, taking as reference the smallest sum LD recorded since the treatment started or the appearance of one or more new lesions
* Stable Disease (SD):	Neither sufficient shrinkage to qualify for PR nor sufficient increase to qualify for PD, taking as reference the smallest sum LD since the treatment started
* Non-progression:	CR + PR + SD but not PD

Hypotheses, clinical

1. A difference in early FDG- or FLT-PET examinations between progressers (PD) and non-progressers (CR, PR, SD) in NSCLC patients treated with erlotinib can be detected.

2. EGFR mutations are found in patients with CR or PR and KRAS mutations in patients with PD after treatment with erlotinib.

Trial design

The trial will be performed as a single-arm, single-center, open-label study.

Recruitment

Patients will be recruited after screening only if all inclusion criteria are met, no exclusion criteria are met and written informed consent has been obtained.

Cancellation of recruitment

The recruitment of patients who are not qualified for the trial can be cancelled. Evidence that the patient is not qualified must be based on data obtained before inclusion (e.g., histology, staging, case history or previous treatment). Only for those patients who were recruited in error without knowledge of these data recruitment can be cancelled. Those for whom a physician later assesses the findings differently or discovers additional involved sites over the course of time (e.g. because of delays in treatment) cannot be cancelled.

7.2 Analysis and trial termination strategy

Interim analyses

No interim analysis is planned.

Early stopping strategy

The trial will be stopped early if unexpectedly high rates of SAEs are observed.

Analyses of primary endpoints

1. The prognostic value/accuracy of FDG-/FLT-PET regarding non-progression in NSCLC patients treated with erlotinib will be evaluated by ROC analysis (area under the ROC curve, AUC); H_0: $AUC_{FDG/FLT\ PET} \leq 0.5$, H_A: $AUC_{FDG/FLT\ PET} > 0.5$ (intersection-union test at one-sided level 2.5% – *primary objective*).

2. The statistical association of EGFR respectively KRAS mutational status, EGFR positivity and clinical response in NSCLC patients treated with erlotinib will be evaluated by Fisher's exact test (at one-sided level 2.5%; strong type 1 error control is maintained if analysis of item 1 was significant – *secondary objective*).

Analysis of secondary endpoints

The prognostic value/accuracy of FDG/FLT-PET regarding clinical failure, SD and clinical response in NSCLC patients treated with erlotinib will be compared by ROC analysis; optimal cut-off values for FDG/FLT PET will be chosen (*secondary objective*).

Association between EGFR mutation, KRAS mutation and EGFR positivity with clinical response will be tested by Fisher´s exact test.

Secondary endpoints (FFTF and OS) will be analyzed using descriptive statistics after at least one year follow-up of all patients.

Statistical Reporting

The final statistical report will be done by PD Dr. M. Hellmich and included also in the final report of the study.

7.3 Further analyses

Subsequent analyses

Expected results

Based on the results of clinical trials with erlotinib in NSCLC patients in Western Europe and the US, we expect about 10% of patients with response (CR, PR) to treatment with erlotinib, about 30% with stable disease and about 60% with progressive disease. Furthermore, we expect about 90% of patients with mutated EGFR responding to therapy. KRAS mutations and EGFR mutations are expected to be nearly exclusive.

Based on published PET data, especially from patients with GIST treated with imatinib, we expect a nearly 100% association of clinical response (CR, PR in CT after 6 weeks of treatment) and reduction in SUV (PET analyses on days 8,9).

Expected flow of information

For the expected patient population similar response rates as described in published studies for Western World patients are expected.

Sample size calculation

Assumptions:

(1) 10% responder, 60% progressers; thus 40% non-progressers

(2) Primary variable is the ‘area under the ROC curve (AUC, ϑ)’, (PET-values of non-progressers > progressers)

(3) Variability in the PET-results of non-progressers is greater than that of progressers

(4) Type-I-error 0.05; type-II-error 0.20

(5) True accuracy (AUC) is at least 0.70 (alternative hypothesis; some common diagnostic tests have an AUC greater than 0.70 [1; p29])

Sample size calculation:

AUC (ϑ)	# Non-progressers	# progressers	# Total
0.70	26	38	64
**0.75**	**16**	**24**	**40**
0.80	11	16	27

Note: The sample size was calculated using formulae (6.3) and (6.6) in [Zhou et al., 2002; p199, 201].

Thus, assuming ϑ_1 = 0.75, 40 patients are needed to reject the null hypothesis H_0: $\vartheta = 0.5$ in favour of the alternative H_1: $\vartheta \neq 0.5$. The sample size will be sufficient to yield a 95% confidence interval for ϑ with width $2 \times 0.17 = 0.34$.

Subgroup analysis

To evaluate differential results concerning the predictive value of the PET analysis between male and female patients a separate analysis for both groups will be performed.

7.4 Data analysis

Raw data of PET will be analyzed by two experienced nuclear physicians in consensus by visual control and semiquantitative (Standardized Uptake Value, SUV) methods using the MPI-tool with knowledge of the CT-findings. Results of the diagnostic procedures will be documented in the CRF on the same day or up to 3 days thereafter latest. Tomograms will

be analyzed in all three planes (coronar, sagital, transversal). Data will be correlated with computed tomography. Data will be stored for direct comparison in the follow-up.

Raw data of CT will be analyzed by two experienced radiologists in consensus according to the RECIST criteria (Therasse *et al.*, 2000) with knowledge of the FDG-PET and FLT-PET. Results of the diagnostic procedures will be documented in the CRF on the same day or up to 3 days thereafter latest.

To assure data quality, an external review of imaging findings will be performed after enrollment of the last patient.

Statistical analysis will be performed by M. Hellmich. The ROC analysis will be performed nonparametrically according to (DeLong et al., 1988).

Inclusion in the analysis

Full analysis set (primary analysis, intention-to-treat): All patients included in the study will be analysed (contingent on availability of endpoints). Per-protocol set (secondary analysis): Only patients fully compliant with the protocol (especially regarding dosage) will be analysed. The full analysis set will be taken as safety analysis population as all patients will receive the study medication.

8 DOCUMENTATION AND MONITORING

All data collected are documented on standardized case report forms (CRF) by the treating physician or other persons given the responsibility for documentation.

A multilevel plan for data validation has been drawn up. In case of missing or inconsistent data, a data manager will contact the treating physician or person responsible for documentation in the department by telephone or in writing (queries).

8.1 Direct access to source data

A regulatory authority may wish to conduct an inspection (during the trial or even after its completion). If an inspection is requested by a regulatory authority, the investigator must inform the trial coordination center immediately that this request has been made. The investigator will permit trial-related monitoring, audits, reviews by the independent ethics committee, and regulatory inspections, providing direct access to source data and documents.

8.2 Quality control and assurance

Each investigator is responsible at his site that the trial is performed in accordance with GCP, AMG and the protocol. He ensures that data is collected as stated in the protocol and

documented correctly in the responding CRF. Monitors will assist investigators in reviewing the complete, legible, clearly arranged and recallable data. All data is examined by the monitors.

8.3 Monitoring

In order to guarantee a high quality of the trial and data retrieval, all participating centers will be visited on a regular base on site by monitors.

Data protection rights will be respected. Patient files will be analyzed on a random base to control original data and to verify accurate data registration and management. The presence of a written informed consent form and the correct interpretation of inclusion and exclusion criteria will be controlled. The monitor will also have regular contact by phone and/or e-mail with all participating centers.

The investigator agrees that the trial center will be visited on a regular base on site by the monitor and will support the monitor in his/her activities.

Purpose of this visits are:

- Evaluation of the trial progression
- Adherence to the trial protocol
- Discussion of problems related to trial problems (adverse events)
- Check of the CRFs concerning accuracy and completeness
- Validation of the CRFs compared to the source data
- Control of the administration of the trial medication

The monitor writes a monitoring-report about each visit. In this monitoring report will be documented the trial progress and all appearing problems.

Data handling and record keeping

Documentation

All protocol required procedures along with information necessary to report the observations and tests described in this protocol will be recorded in the Case report forms (CRFs). CRFs must be completed and given to the trial coordination center in a timely and accurate manner by site personnel.

CT scans must be reported in summary in the CRF. The original reports, traces and films must be retained by the investigator for future reference.

The investigator must review all pages within the CRF for accuracy and consistency with the protocol, and sign and date the CRF sign-off page(s) upon completion.

Corrections to data on the CRFs must be made by lining out the incorrect data with a single line and writing the correct data near to those crossed out. Correction fluid is not to be used

to cover errors. Each correction must be initialed and dated by the person making the correction.

The patient consent forms designated for the clinical investigator are also to be kept in the trial documentation. All the information on which the entries in the CRF are based must be available in the patient files e. g. results of laboratory investigations.

After prior agreement, a check of the consistency of data between the patient files, raw data and CRF as well as with other documents related to the trial will be conducted by the responsible authorities and/or by monitors (inspection/audit/monitoring).

The clinical investigator is responsible for the identity of the data in the patient file and the correct entry of the data into the CRF.

Data management

All incoming CRF and DCF will be registered by the data management. All registration forms will be checked for completeness, plausibility, and correctness. Missing, questionable, or additional information will be requested on a regular base by the data management. The data management will remind for missing documentation regularly.

Data will be entered into an Oracle database using MACRO as the data entry application. During entry data will be checked by constraints and triggers.

The database will be checked for faults and validated by the database programmer. Thereafter the database will approve the database for data entry. Complete and incremental data backup will be performed regularly.

All CRFs and other essential trial documents will be archived for at least 15 years. Patients ID-lists and medical records will be kept separately at the individual trial sites.

Record keeping

A copy of the final completed CRFs is retained by the investigator, who must ensure that it is stored with other trial documents, such as the protocol, the investigator's brochure and any protocol amendments, in a secure place.

Case records and raw data are to be retained for at least 15 years following the end of the trial. The investigator will ensure that a correct assignment of the CRFs to the corresponding patient files and raw data is possible at any time. A separate patient identification list must be recorded by the investigator. Patients ID-lists and medical records will be kept separately at the individual trial sites by the investigator.

9 ETHICAL MATTERS

The trial is performed according to the declaration of Helsinki (Helsinki, 2000) and the principles of the ICH guidelines on Good Clinical Practice (Spira and Ettinger, 2004). The ICH guidelines have been adapted to the conditions applying to quality assurance protocols in clinical trials groups.

The trial coordinators have obtained a vote of approval from the local responsible ethical committee - (Ethikkommission der Universität zu Köln) of the responsible authorities (BfArM and BFS) according to the German drug regulations (Arzneimittelgesetzt, 2004).

For PET diagnostics, the trial coordinators will obtain the approval of the German Authority for Radiation Safety (Bundesamt für Strahlenschutz). As synthesis and application of FLT and FDG is performed within the trial center, §4a.3 of the AMG is applicable.

Other radiation burdens (radiotherapy, X-rays) lie within the range of usual medical care and approval is therefore, under present legislation, not required.

The trial coordinators will obtain approvement the responsible ethical committee, according to the German drug regulations, whenever the trial protocol is amended such as to require renewed assessment of risk to patients (e.g. changes to dose or to duration of treatment, or the occurrence of serious adverse events).

Roche will fulfill reporting requirements to authorities and ethics committees. SUSARs will be distributed to ethics committees and centers by Roche.

10 ORGANIZATION

10.1 Retrospective protocol amendments

Protocol amendments or additions can be agreed upon by the trial coordinators on the basis of current information in the literature for reasons of ethics or safety.

Any protocol amendment is to be:

- Approved by the ethical committee and the regulatory authority if the risk to patients must be newly assessed;
- recorded in the protocol logbook, with date

10.2 Insurance

Insurance (Gerling) is provided for the patients in this trial. Details can be obtained from the trial coordination center.

10.3 Agreement on publication

Analyses and publications (lectures, articles, etc.) of data and information connected with the present quality assurance protocol may be made solely with the agreement of the LCGC trial coordinator. Any formal presentation or publication of data collected from this trial will be considered as a joint publication by the investigators, the principal investigators, and the sponsor. Authorship will be determined by mutual agreement.

11 REFERENCES

Arzneimittelgesetzt (2004). *Bundesgesetzblatt* **Version of 30 July**.

Barge A. (2004).

Barthel H, Cleij M, Collingridge D, Hutchinson O, Osman S, He Q *et al* (2003). 3'-deoxy-3'-[18F]fluorothymidine as a new marker for monitoring tumor response to antiproliferative therapy in vivo with positron emission tomography. *Cancer Res* **63:** 3791-8.

Buck AK, Halter G, Schirrmeister H, Kotzerke J, Wurziger I, Glatting G *et al* (2003). Imaging proliferation in lung tumors with PET: 18F-FLT versus 18F-FDG. *J Nucl Med* **44:** 1426-31.

Buck AK, Schirrmeister H, Hetzel M, Von Der Heide M, Halter G, Glatting G *et al* (2002). 3-deoxy-3-[(18)F]fluorothymidine-positron emission tomography for noninvasive assessment of proliferation in pulmonary nodules. *Cancer Res* **62:** 3331-4.

Cappuzzo F, Hirsch FR, Rossi E, Bartolini S, Ceresoli GL, Bemis L *et al* (2005). Epidermal growth factor receptor gene and protein and gefitinib sensitivity in non-small-cell lung cancer. *J Natl Cancer Inst* **97:** 643-55.

Charnley NG WC, Barnett C, Brock CS, Bydder GM, Glaser M, Newlands ES, Price P (2005). Early change in glucose metabolic rate (MRGlu) measured using FDG-PET in patients with high grade glioma (HGG) and correlation with response to temozolomide, and temozolomide + radiotherapy. *ASCO Annual Meeting***:** Abstract No: 1532.

Cobleigh MA, Vogel CL, Tripathy D, Robert NJ, Scholl S, Fehrenbacher L *et al* (1999). Multinational study of the efficacy and safety of humanized anti-HER2 monoclonal antibody in women who have HER2-overexpressing metastatic breast cancer that has progressed after chemotherapy for metastatic disease. *J Clin Oncol* **17:** 2639-48.

D'Addario G, Pintilie M, Leighl NB, Feld R, Cerny T, Shepherd FA (2005). Platinum-based versus non-platinum-based chemotherapy in advanced non-small-cell lung cancer: a meta-analysis of the published literature. *J Clin Oncol* **23:** 2926-36.

DeLong ER, DeLong DM, Clarke-Pearson DL (1988). Comparing the areas under two or more correlated receiver operating characteristic curves: a nonparametric approach. *Biometrics* **44:** 837-45.

Di Fabio F, Pinto, C, Fanti, S, Ceccarelli, C, Gentile, AL, Rojas Llimpe, FL, Nanni, C, Mutri, V, Cacciari, N, Martoni, A. (2005). CORRELATION BETWEEN FDG-PET AND PATHOLOGIC RESPONSE IN PATIENTS WITH RECTAL CANCER TREATED WITH NEOADJUVANT CHEMO-RADIOTHERAPY: FIRST RESULTS OF THE BOLOGNA PROJECT. *ASCO Annual Meeting***:** Abstract No: 3623.

Druker BJ, Sawyers CL, Kantarjian H, Resta DJ, Reese SF, Ford JM *et al* (2001). Activity of a specific inhibitor of the BCR-ABL tyrosine kinase in the blast crisis of

chronic myeloid leukemia and acute lymphoblastic leukemia with the Philadelphia chromosome. *N Engl J Med* **344:** 1038-42.

Fukuoka M, Yano S, Giaccone G, Tamura T, Nakagawa K, Douillard JY *et al* (2003). Multi-institutional randomized phase II trial of gefitinib for previously treated patients with advanced non-small-cell lung cancer (The IDEAL 1 Trial) [corrected]. *J Clin Oncol* **21:** 2237-46.

Gatzemeier U, Pluzanska, A., Szczesna, A., Kaukel, E., Roubec, J., Brennscheidt, U., De Rosa, F., Mueller, B., Von Pawel, J., (2004). Results of a phase III trial of erlotinib (OSI-774) combined with cisplatin and gemcitabine (GC) chemotherapy in advanced non-small cell lung cancer (NSCLC). *Journal of Clinical Oncology, 2004 ASCO Annual Meeting Proceedings (Post-Meeting Edition).* **22:** 7010.

Gatzemeier U, Rosell, R., Ramlau, R., Robinet, G., Szczesna, A., Quoix, E., Font, A., Jimenez, E., Mueser, M., Harstrick, A. (2003). *ASCO, Vol. 22*. Oncol PASC (ed.), pp 2582.

Giaccone G, Lechevalier, T., Thatcher, N., Smit, E., Janmaat, M., Rodriguez, J., Oulid-Aissa, D., Soria, J.C. (2005). *ASCO*, pp Abstract No. 7073.

Griesinger F, Overbeck,T, Dörge, H, Schmidberger, H, Meller, J, Andreas, S, Keppler, U, Schirren, J, Hemmerlein, B, Baum, RP (2005). Phase II induction therapy with docetaxel and carboplatin in NSCLC IIIA/IIIB: FDG-PET Response Predicts Overall and Disease Free Survival. *ASCO Annual Meeting***:** Abstract No: 7184.

Hanna N, Shepherd FA, Fossella FV, Pereira JR, De Marinis F, von Pawel J *et al* (2004). Randomized phase III trial of pemetrexed versus docetaxel in patients with non-small-cell lung cancer previously treated with chemotherapy. *J Clin Oncol* **22:** 1589-97.

Helsinki Do (2000). Ethical principles for medical research involving human subjects. *Adopted June 1969, last amendment 2000* **http://fasels.org/arvo/helsinki.htm**.

Herbst RS, Prager, D., Hermann, R., Miller, V., Fehrenbacher, L., Hoffman, P., Johnson, B., Sandler, A.B., Mass, R., Johnson, D.H, (2004). TRIBUTE - A phase III trial of erlotinib HCl (OSI-774) combined with carboplatin and paclitaxel (CP) chemotherapy in advanced non-small cell lung cancer (NSCLC). *Journal of Clinical Oncology, 2004 ASCO Annual Meeting Proceedings (Post-Meeting Edition).* **22:** 7011.

Jackman DM, Yeap BY, Lindeman NI, Fidias P, Rabin MS, Temel J *et al* (2007). Phase II clinical trial of chemotherapy-naive patients > or = 70 years of age treated with erlotinib for advanced non-small-cell lung cancer. *J Clin Oncol* **25:** 760-6.

Jager PL, Gietema JA, van der Graaf WT (2004). Imatinib mesylate for the treatment of gastrointestinal stromal tumours: best monitored with FDG PET. *Nucl Med Commun* **25:** 433-8.

Jemal A, Tiwari RC, Murray T, Ghafoor A, Samuels A, Ward E *et al* (2004). Cancer statistics, 2004. *CA Cancer J Clin* **54:** 8-29.

Johnson B, Lucca, J, Rabin, MS (2004). A phase II trial of erlotinib in previuosly untreated elderly patients (> 70 years) with advanced NSCLC: Preliminary results. *Ann Oncol* **15:** iii169.

Kimura T, Kudoh, S, Yoshimura, N, Mitsuoka, S, Matsuura, K, Koyama, K, Kawabe, J, Okamura, M, Hirata, K, Shiomi, S, Yoshikawa, J. (2005). Monitoring of chemotherapy response in multiple disseminated malignant pleural mesothelioma using fluorodeoxyglucose positron emission tomography. *ASCO Annual Meeting***:** Abstract No: 7177.

Kris MG, Natale RB, Herbst RS, Lynch TJ, Jr., Prager D, Belani CP *et al* (2003). Efficacy of gefitinib, an inhibitor of the epidermal growth factor receptor tyrosine kinase, in symptomatic patients with non-small cell lung cancer: a randomized trial. *Jama* **290:** 2149-58.

L Bastholt LS, K Jensen, E Brun, A Loft, J Petersen, H Kastberg, JG Eriksen. (2005). *ASCO Annual Meeting*, pp Abstract No: 5530.

Leyton J, Latigo J, Perumal M, Dhaliwal H, He Q, Aboagye E (2005). Early detection of tumor response to chemotherapy by 3'-deoxy-3'-[18F]fluorothymidine positron emission tomography: the effect of cisplatin on a fibrosarcoma tumor model in vivo. *Cancer Res* **65:** 4202-10.

Lynch TJ, Bell DW, Sordella R, Gurubhagavatula S, Okimoto RA, Brannigan BW *et al* (2004). Activating mutations in the epidermal growth factor receptor underlying responsiveness of non-small-cell lung cancer to gefitinib. *N Engl J Med* **350:** 2129-39.

Paez JG, Janne PA, Lee JC, Tracy S, Greulich H, Gabriel S *et al* (2004). EGFR mutations in lung cancer: correlation with clinical response to gefitinib therapy. *Science* **304:** 1497-500.

Pao W, Miller V, Zakowski M, Doherty J, Politi K, Sarkaria I *et al* (2004). EGF receptor gene mutations are common in lung cancers from "never smokers" and are associated with sensitivity of tumors to gefitinib and erlotinib. *Proc Natl Acad Sci U S A* **101:** 13306-11.

Pao W, Miller VA, Politi KA, Riely GJ, Somwar R, Zakowski MF *et al* (2005). Acquired resistance of lung adenocarcinomas to gefitinib or erlotinib is associated with a second mutation in the EGFR kinase domain. *PLoS Med* **2:** e73.

Patel J, Miller, VA, Kris MG (2003). Encouraging activity and durable response demonstrated by the EGFR tyrosine kinase inhibitor erlotinib (Tarceva TM, OSI-774) in patients with advanced bronchioalveolar (BAC) cell carcinoma. *Lung Cancer* **41 (Suppl.):** S56.

Perez-Soler R (2004). Phase II clinical trial data with the epidermal growth factor receptor tyrosine kinase inhibitor erlotinib (OSI-774) in non-small-cell lung cancer. *Clin Lung Cancer* **6 Suppl 1:** S20-3.

Perez-Soler R, Chachoua A, Hammond LA, Rowinsky EK, Huberman M, Karp D *et al* (2004). Determinants of tumor response and survival with erlotinib in patients with non--small-cell lung cancer. *J Clin Oncol* **22:** 3238-47.

Pfister DG, Johnson DH, Azzoli CG, Sause W, Smith TJ, Baker S, Jr. *et al* (2004). American Society of Clinical Oncology treatment of unresectable non-small-cell lung cancer guideline: update 2003. *J Clin Oncol* **22:** 330-53.

Pieterman RM, van Putten JW, Meuzelaar JJ, Mooyaart EL, Vaalburg W, Koeter GH *et al* (2000). Preoperative staging of non-small-cell lung cancer with positron-emission tomography. *N Engl J Med* **343:** 254-61.

Reck M, Buchholz E, Romer KS, Krutzfeldt K, Gatzemeier U, Manegold C (2006). Gefitinib monotherapy in chemotherapy-naive patients with inoperable stage III/IV non-small-cell lung cancer. *Clin Lung Cancer* **7:** 406-11.

Rodenhuis S, Slebos RJ (1990). The ras oncogenes in human lung cancer. *Am Rev Respir Dis* **142:** S27-30.

Rodenhuis S, Slebos RJ, Boot AJ, Evers SG, Mooi WJ, Wagenaar SS *et al* (1988). Incidence and possible clinical significance of K-ras oncogene activation in adenocarcinoma of the human lung. *Cancer Res* **48:** 5738-41.

Rousseau C, Ferrer, L, Bridji, B, Campion, L, Sagan, C, Ricaud,M, Resche, I, Fleury,N, Campone, M. (2005). EARLY RESPONSE MONITORING BY FDG-PET TO NEOADJUVANT CHEMOTHERAPY IN LOCALLY ADVANCED BREAST CANCER PATIENTS. *ASCO Annual Meeting***:** Abstract No: 2022.

Sandler A, Gray, R, Brahmer, J, Dowlati, A, Schiller, JH, Perry, MC, Johnson, DH. (2005). Randomized phase II/III trial of paclitaxel (P) plus carboplatin (C) with or without bevacizumab (NSC #704865) in patients with advanced non-squamous non-small cell lung cancer (NSCLC): An Eastern Cooperative Oncology Group (ECOG) Trial - E4599. *J Clin Oncol* **23:** 1090s.

Sankhala K, Chawla, SP, Iagaru, A, Dellamaggiora, R, Chua, V, Daly, S, Bedrosian, CL, Edwards, GK, Cohen, S, Demetri, GD for the AP23573 Sarcoma Study Group (2005). Early response evaluation of therapy with AP23573 (an mTOR inhibitor) in sarcoma using [18F]2-fluoro-2-deoxy-D-glucose (FDG) positron emission tomography (PET) scan. *ASCO Annual Meeting***:** Abstract No: 9028.

Schiller JH, Harrington D, Belani CP, Langer C, Sandler A, Krook J *et al* (2002). Comparison of four chemotherapy regimens for advanced non-small-cell lung cancer. *N Engl J Med* **346:** 92-8.

Schmuecking M, Schneider, CP, Soeldner, J, Leonhardi, J, Presselt, N, Scheithauer, M, Baum, RP, Mueller, KM, Wendt, TG, Bonnet, R. (2005). What is the role of F-18 FDG PET within randomized multicenter clinical trials for multi-modality treatment of non-small cell lung cancer stage III? *ASCO Annual Meeting***:** Abstract No: 7182.

Schuetze S, Eary, JF, Griffith, KA, Rubin, BP, Hawkins, DS, Vernon, CB, Mann, GN, Conrad, EU. (2005). FDG PET but not RECIST agrees with histologic response of soft

tissue sarcoma to neoadjuvant chemotherapy. *ASCO Annual Meeting*: Abstract No: 9005.

Shepherd FA, Dancey J, Ramlau R, Mattson K, Gralla R, O'Rourke M *et al* (2000). Prospective randomized trial of docetaxel versus best supportive care in patients with non-small-cell lung cancer previously treated with platinum-based chemotherapy. *J Clin Oncol* **18:** 2095-103.

Shepherd FA, Rodrigues Pereira J, Ciuleanu T, Tan EH, Hirsh V, Thongprasert S *et al* (2005). Erlotinib in previously treated non-small-cell lung cancer. *N Engl J Med* **353:** 123-32.

Shields AF, Grierson JR, Dohmen BM, Machulla HJ, Stayanoff JC, Lawhorn-Crews JM *et al* (1998). Imaging proliferation in vivo with [F-18]FLT and positron emission tomography. *Nat Med* **4:** 1334-6.

Spira A, Ettinger DS (2004). Multidisciplinary management of lung cancer. *N Engl J Med* **350:** 379-92.

Stroobants S, Goeminne J, Seegers M, Dimitrijevic S, Dupont P, Nuyts J *et al* (2003). 18FDG-Positron emission tomography for the early prediction of response in advanced soft tissue sarcoma treated with imatinib mesylate (Glivec). *Eur J Cancer* **39:** 2012-20.

Sugiyama S (2004). [Chronic rejection and chronic allograft nephropathy]. *Nippon Jinzo Gakkai Shi* **46:** 667-75.

Suzuki Y, Orita M, Shiraishi M, Hayashi K, Sekiya T (1990). Detection of ras gene mutations in human lung cancers by single-strand conformation polymorphism analysis of polymerase chain reaction products. *Oncogene* **5:** 1037-43.

Therasse P, Arbuck SG, Eisenhauer EA, Wanders J, Kaplan RS, Rubinstein L *et al* (2000). New guidelines to evaluate the response to treatment in solid tumors. European Organization for Research and Treatment of Cancer, National Cancer Institute of the United States, National Cancer Institute of Canada. *J Natl Cancer Inst* **92:** 205-16.

Tsao M, Sakurada, A, Lorimer, I, Cutz, J, Kamel-Reid, S, Squire, J, Ding, K, Frank, R, Seymour, L, Shepherd, F. (2005). Molecular analysis of the epidermal growth factor receptor (EGFR) gene and protein expression in patients treated with erlotinib in National Cancer Institute of Canada Clinical Trials Group (NCIC CTG) trial BR.21. *ASCO Annual Meeting*: Abstract No: 7007.

van den Abbeele A, Melenevsky, Y, de Vries, D, Manola, J, Dileo, P, Tetrault, R, Baum, C, Badawi, R, Demetri, G. (2005). Imaging kinase target inhibition with SU11248 by FDG-PET in patients (pts) with imatinib-resistant gastrointestinal stromal tumors (I-R GIST). *ASCO Annual Meeting*: Abstract No: 9006.

van Tinteren H, Hoekstra OS, Smit EF, van den Bergh JH, Schreurs AJ, Stallaert RA *et al* (2002). Effectiveness of positron emission tomography in the preoperative

assessment of patients with suspected non-small-cell lung cancer: the PLUS multicentre randomised trial. *Lancet* **359:** 1388-93.

Yarden Y (2001). The EGFR family and its ligands in human cancer. signalling mechanisms and therapeutic opportunities. *Eur J Cancer* **37 Suppl 4:** S3-8.

12 APPENDIX

12.1 Copies of legal documents

Declaration of Helsinki

WORLD MEDICAL ASSOCIATION DECLARATION OF HELSINKI

Ethical Principles for Medical Research Involving Human Subjects

Adopted by the 18th WMA General Assembly
Helsinki, Finland, June 1964
and amended by the
29th WMA General Assembly, Tokyo, Japan, October 1975
35th WMA General Assembly, Venice, Italy, October 1983
41st WMA General Assembly, Hong Kong, September 1989
48th WMA General Assembly, Somerset West, Republic of South Africa, October 1996
and the
52nd WMA General Assembly, Edinburgh, Scotland, October 2000
Note of Clarification on Paragraph 29 added by the WMA General Assembly,
Washington 2002

A. INTRODUCTION

1. The World Medical Association has developed the Declaration of Helsinki as a statement of ethical principles to provide guidance to physicians and other participants in medical research involving human subjects. Medical research involving human subjects includes research on identifiable human material or identifiable data.

2. It is the duty of the physician to promote and safeguard the health of the people. The physician's knowledge and conscience are dedicated to the fulfillment of this duty.

3. The Declaration of Geneva of the World Medical Association binds the physician with the words, "The health of my patient will be my first consideration," and the International Code of Medical Ethics declares that, "A physician shall act only in the patient's interest when providing medical care which might have the effect of weakening the physical and mental condition of the patient."

4. Medical progress is based on research which ultimately must rest in part on experimentation involving human subjects.

5. In medical research on human subjects, considerations related to the well-being of the human subject should take precedence over the interests of science and society.

6. The primary purpose of medical research involving human subjects is to improve prophylactic, diagnostic and therapeutic procedures and the understanding of the aetiology and pathogenesis of disease. Even the best proven prophylactic, diagnostic, and therapeutic methods must continuously be challenged through research for their effectiveness, efficiency, accessibility and quality.

7. In current medical practice and in medical research, most prophylactic, diagnostic and therapeutic procedures involve risks and burdens.

8. Medical research is subject to ethical standards that promote respect for all human beings and protect their health and rights. Some research populations are vulnerable and need special protection. The particular needs of the economically and medically disadvantaged must be recognized. Special attention is also required for those who cannot give or refuse consent for themselves, for those who may be subject to giving consent under duress, for those who will not benefit personally from the research and for those for whom the research is combined with care.

9. Research Investigators should be aware of the ethical, legal and regulatory requirements for research on human subjects in their own countries as well as applicable international requirements. No national ethical, legal or regulatory requirement should be allowed to reduce or eliminate any of the protections for human subjects set forth in this Declaration.

B. BASIC PRINCIPLES FOR ALL MEDICAL RESEARCH

10. It is the duty of the physician in medical research to protect the life, health, privacy, and dignity of the human subject.

11. Medical research involving human subjects must conform to generally accepted scientific principles, be based on a thorough knowledge of the scientific literature, other relevant sources of information, and on adequate laboratory and, where appropriate, animal experimentation.

12. Appropriate caution must be exercised in the conduct of research which may affect the environment, and the welfare of animals used for research must be respected.

13. The design and performance of each experimental procedure involving human subjects should be clearly formulated in an experimental protocol. This protocol should be submitted for consideration, comment, guidance, and where appropriate, approval to a specially appointed ethical review committee, which must be independent of the investigator, the sponsor or any other kind of undue influence. This independent committee should be in conformity with the laws and regulations of the country in which the research experiment is performed. The committee has the right to monitor ongoing trials. The researcher has the obligation to provide monitoring information to the committee, especially any serious adverse events. The researcher should also submit to the committee, for review, information regarding funding, sponsors, institutional affiliations, other potential conflicts of interest and incentives for subjects.

14. The research protocol should always contain a statement of the ethical considerations involved and should indicate that there is compliance with the principles enunciated in this Declaration.

15. Medical research involving human subjects should be conducted only by scientifically qualified persons and under the supervision of a clinically competent medical person. The responsibility for the human subject must always rest with a medically qualified person and never rest on the subject of the research, even though the subject has given consent.

16. Every medical research project involving human subjects should be preceded by careful assessment of predictable risks and burdens in comparison with foreseeable benefits to the subject or to others. This does not preclude the participation of healthy volunteers in medical research. The design of all studies should be publicly available.

17. Physicians should abstain from engaging in research projects involving human subjects unless they are confident that the risks involved have been adequately assessed and can be satisfactorily managed. Physicians should cease any investigation if the risks are found to outweigh the potential benefits or if there is conclusive proof of positive and beneficial results.

18. Medical research involving human subjects should only be conducted if the importance of the objective outweighs the inherent risks and burdens to the subject. This is especially important when the human subjects are healthy volunteers.

19. Medical research is only justified if there is a reasonable likelihood that the populations in which the research is carried out stand to benefit from the results of the research.

20. The subjects must be volunteers and informed participants in the research project.

21. The right of research subjects to safeguard their integrity must always be respected. Every precaution should be taken to respect the privacy of the subject, the confidentiality of the patient's information and to minimize the impact of the study on the subject's physical and mental integrity and on the personality of the subject.

22. In any research on human beings, each potential subject must be adequately informed of the aims, methods, sources of funding, any possible conflicts of interest, institutional affiliations of the researcher, the anticipated benefits and potential risks of the study and the discomfort it may entail. The subject should be informed of the right to abstain from participation in the study or to withdraw consent to participate at any time without reprisal. After ensuring that the subject has understood the information, the physician should then obtain the subject's freely-given informed consent, preferably in writing. If the consent cannot be obtained in writing, the non-written consent must be formally documented and witnessed.

23. When obtaining informed consent for the research project the physician should be particularly cautious if the subject is in a dependent relationship with the physician or may consent under duress. In that case the informed consent should be obtained by a well-informed physician who is not engaged in the investigation and who is completely independent of this relationship.

24. For a research subject who is legally incompetent, physically or mentally incapable of giving consent or is a legally incompetent minor, the investigator must obtain informed consent from the legally authorized representative in accordance with applicable law. These groups should not be included in research unless the research is necessary to promote the health of the population represented and this research cannot instead be performed on legally competent persons.

25. When a subject deemed legally incompetent, such as a minor child, is able to give assent to decisions about participation in research, the investigator must obtain that assent in addition to the consent of the legally authorized representative.

26. Research on individuals from whom it is not possible to obtain consent, including proxy or advance consent, should be done only if the physical/mental condition that prevents obtaining informed consent is a necessary characteristic of the research population. The specific reasons for involving research subjects with a condition that renders them unable to give informed consent should be stated in the experimental protocol for consideration and approval of the review committee. The protocol should state that consent to remain in the research should be obtained as soon as possible from the individual or a legally authorized surrogate.

27. Both authors and publishers have ethical obligations. In publication of the results of research, the investigators are obliged to preserve the accuracy of the results. Negative as well as positive results should be published or otherwise publicly available. Sources of funding, institutional affiliations and any possible conflicts of interest should be declared in the publication. Reports of experimentation not in accordance with the principles laid down in this Declaration should not be accepted for publication.

C. ADDITIONAL PRINCIPLES FOR MEDICAL RESEARCH COMBINED WITH MEDICAL CARE

28. The physician may combine medical research with medical care, only to the extent that the research is justified by its potential prophylactic, diagnostic or therapeutic value. When medical research is combined with medical care, additional standards apply to protect the patients who are research subjects.

29. The benefits, risks, burdens and effectiveness of a new method should be tested against those of the best current prophylactic, diagnostic, and therapeutic methods. This does not exclude the use of placebo, or no treatment, in studies where no proven prophylactic, diagnostic or therapeutic method exists. See footnote

30. At the conclusion of the study, every patient entered into the study should be assured of access to the best proven prophylactic, diagnostic and therapeutic methods identified by the study.

31.The physician should fully inform the patient which aspects of the care are related to the research. The refusal of a patient to participate in a study must never interfere with the patient-physician relationship.

32. In the treatment of a patient, where proven prophylactic, diagnostic and therapeutic methods do not exist or have been ineffective, the physician, with informed consent from the patient, must be free to use unproven or new prophylactic, diagnostic and therapeutic measures, if in the physician's judgement it

offers hope of saving life, re-establishing health or alleviating suffering. Where possible, these measures should be made the object of research, designed to evaluate their safety and efficacy. In all cases, new information should be recorded and, where appropriate, published. The other relevant guidelines of this Declaration should be followed.

FOOTNOTE: NOTE OF CLARIFICATION ON PARAGRAPH 29 of the WMA DECLARATION OF HELSINKI

The WMA hereby reaffirms its position that extreme care must be taken in making use of a placebo-controlled trial and that in general this methodology should only be used in the absence of existing proven therapy. However, a placebo-controlled trial may be ethically acceptable, even if proven therapy is available, under the following circumstances:

- Where for compelling and scientifically sound methodological reasons its use is necessary to determine the efficacy or safety of a prophylactic, diagnostic or therapeutic method; or

- Where a prophylactic, diagnostic or therapeutic method is being investigated for a minor condition and the patients who receive placebo will not be subject to any additional risk of serious or irreversible harm.

All other provisions of the Declaration of Helsinki must be adhered to, especially the need for appropriate ethical and scientific review.

The Declaration of Helsinki (Document 17.C) is an official policy document of the World Medical Association, the global representative body for physicians. It was first adopted in 1964 (Helsinki, Finland) and revised in 1975 (Tokyo, Japan), 1983 (Venice, Italy), 1989 (Hong Kong), 1996 (Somerset-West, South Africa) and 2000 (Edinburgh, Scotland). Note of clarification on Paragraph 29 added by the WMA General Assembly,
Washington 2002.

Vote of approval by the ethical committee of Universitätsklinikum Köln (original)

The ERLOPET clinical study protocol is currently submitted to the ethics committee of the University Hospital of Cologne.

Confirmation of insurance

The confirmation of insurance will be added after the final vote of the ethics committee of the University Hospital of Cologne.

12.2 Addresses

Responsible persons

Principal Investigator:

Prof. Dr. Jürgen Wolf, Department I of Internal Medicine, University Clinic Cologne, Kerpenerstr. 62, 50924 Cologne
Tel: +49-(0)2 21-4 78-44 89 / Fax: +49-(0) 2 21-4 78-67 33

Co-investigators:

Dr. Thomas Zander, Dr. Roman Thomas, Dr. Karin Töpelt, Dr. Lucia Nogová, Matthias Scheffler, Department I of Internal Medicine, University Clinic Cologne, Kerpenerst. 62, 50924 Cologne
Tel: +49-(0)2 21-4 78-54 13 / 34 10 / Fax: +49-(0) 2 21-4 78-36 47

PET:

Prof. Dr. Harald Schicha, PD Dr. Markus Dietlein, Department Nuclear Medicine, University Clinic Cologne, Kerpenerstr. 62, 50924 Cologne
Tel: +49-(0)2 21-4 78-40 50 / 58 56 / Fax: +49-(0) 2 21-4 78-43 95

Prof. Dr. Andreas Jacobs, Department for Neurology, University Clinic Cologne, Kerpenerstr. 62, 50924 Cologne
Tel: +49-(0)2 21-4 78-36 50 / Fax: +49-(0) 2 21-4 78-70 07

Radiology:

Dr. Christopher Bangard, Institute for Radiology, University Clinic, Kerpenerstr. 62, 50924 Cologne
Tel: +49-(0)2 21-4 78-50 60 / Fax: +49-(0) 2 21-4 78-42 13

Pathology:

PD Dr. Claudia Wickenhauser, Institute for Pathology, University Clinic Cologne, Kerpenerstr. 62, 50924 Cologne
Tel: +49-(0)2 21-4 78-63 20 / Fax: +49-(0) 2 21-4 78-63 60

Statistics:

Dr. Martin Hellmich, Institut für Medizinische Statistik, Informatik und Epidemiologie, University Clinic Cologne, Kerpenerstr. 62, 50924 Cologne
Tel: +49-(0)2 21-4 78-65 09 / Fax: +49-(0) 2 21-4 78-65 20

Monitoring / Datamanagement
ZKS, University Cologne, Gleueler Str. 269, 50935 Cologne

State authority Bundesinstitut für Arzneimittel und Medizinprodukte
Kurt-Georg-Kiesinger-Allee 3
53175 Bonn
Tel.: +49 (0)228 207-30 (Zentrale)
Fax: +49 (0)228 207-5207
E-Mail: poststelle@bfarm.de

12.3 Definitions

Activity index according to the ECOG

The ECOG activity index assesses the performance status of the patient with regard to physical and social factors:

0	Fully active, able to carry on all pre-disease activities without restrictions.
1	Restricted in physically strenuous activity but ambulatory and able to carry out work of a light or sedentary nature e.g. light house work, office work.
2	Ambulatory and capable of all self care but unable to carry out any work activities. Up and about more than 50% of waking hours.
3	Capable of only limited self care, confined to bed or chair more than 50% of waking hours.
4	Completely disabled. Cannot carry on any self care. Totally confined to bed or chair.

Stage of disease

The tumor, node, and metastasis (TNM) staging system for lung cancer, developed by the American Joint Committee on Cancer, is shown in the table below.

Stage	Tumor	Node	Metastasis	General Description
Local				
IA	T1	N0	M0	T1 tumor ≤ 3 cm, surrounded by lung or pleura; no tumor more proximal than lobe bronchus
IB	T2	N0	M0	T2 tumor > 3 cm, involving main bronchus ≥ 2 cm distal to carina, invading pleura; atelectasis or pneumonitis extending to hilum but not entire lung
IIA	T1	N1	M0	N1: involvement of ipsilateral peribronchial or hilar nodes and intrapulmonary nodes by direct extension
Locally advanced				
IIB	T2	N1	M0	
	T3	N0	M0	T3 tumor: invasion of chest wall, diaphragm, mediastinal pleura, pericardium, main bronchus < 2 cm distal to carina; atelectasis or pneumonitis of entire lung.
IIIA	T1	N2	M0	
	T2	N2	M0	
	T3	N1	M0	
	T3	N2	M0	N2: involvement of ipsilateral mediastinal or subcarinal nodes
IIIB	Any T	N3	M0	N3: involvement of contralateral (lung) nodes or any supraclavicular node
Advanced				
IIIB	T4	Any N	M0	T4 Tumor: invasion of mediastinum, heart, great vessels, trachea, esophagus, vertebral body, carina; separate tumor nodules; malignant pleural effusion
IV	Any T	Any N	M1	Distant metastasis

Response Evaluation Criteria in Solid Tumors (RECIST) Quick Reference

Eligibility (http://www3.cancer.gov/bip/RECIST.htm)

- Only patients with measurable disease at baseline should be included in protocols where objective tumor response is the primary endpoint.

  Measurable disease - the presence of at least one measurable lesion. If the measurable disease is restricted to a solitary lesion, its neoplastic nature should be confirmed by cytology/histology.

  Measurable lesions - lesions that can be accurately measured in at least one dimension with longest diameter ≥20 mm using conventional techniques or ≥10 mm with spiral CT scan.

  Non-measurable lesions - all other lesions, including small lesions (longest diameter <20 mm with conventional techniques or <10 mm with spiral CT scan), i.e., bone lesions, leptomeningeal disease, ascites, pleural/pericardial effusion, inflammatory breast disease, lymphangitis cutis/pulmonis, cystic lesions, and also abdominal masses that are not confirmed and followed by imaging techniques; and.

- All measurements should be taken and recorded in metric notation, using a ruler or calipers. All baseline evaluations should be performed as closely as possible to the beginning of treatment and never more than 4 weeks before the beginning of the treatment.

- The same method of assessment and the same technique should be used to characterize each identified and reported lesion at baseline and during follow-up.

- Clinical lesions will only be considered measurable when they are superficial (e.g., skin nodules and palpable lymph nodes). For the case of skin lesions, documentation by color photography, including a ruler to estimate the size of the lesion, is recommended.

Methods of Measurement

- CT and MRI are the best currently available and reproducible methods to measure target lesions selected for response assessment. Conventional CT and MRI should be performed with cuts of 10 mm or less in slice thickness contiguously. Spiral CT should be performed using a 5 mm contiguous reconstruction algorithm. This applies to tumors of the chest, abdomen and pelvis. Head and neck tumors and those of extremities usually require specific protocols.
- Lesions on chest X-ray are acceptable as measurable lesions when they are clearly defined and surrounded by aerated lung. However, CT is preferable.
- When the primary endpoint of the study is objective response evaluation, ultrasound (US) should not be used to measure tumor lesions. It is, however, a possible alternative to clinical measurements of superficial palpable lymph nodes, subcutaneous lesions and

thyroid nodules. US might also be useful to confirm the complete disappearance of superficial lesions usually assessed by clinical examination.

- The utilization of endoscopy and laparoscopy for objective tumor evaluation has not yet been fully and widely validated. Their uses in this specific context require sophisticated equipment and a high level of expertise that may only be available in some centers. Therefore, the utilization of such techniques for objective tumor response should be restricted to validation purposes in specialized centers. However, such techniques can be useful in confirming complete pathological response when biopsies are obtained.
- Tumor markers alone cannot be used to assess response. If markers are initially above the upper normal limit, they must normalize for a patient to be considered in complete clinical response when all lesions have disappeared.
- Cytology and histology can be used to differentiate between PR and CR in rare cases (e.g., after treatment to differentiate between residual benign lesions and residual malignant lesions in tumor types such as germ cell tumors).

Baseline documentation of "Target" and "Non-Target" lesions

- All measurable lesions up to a maximum of five lesions per organ and 10 lesions in total, representative of all involved organs should be identified as ***target lesions*** and recorded and measured at baseline.
- Target lesions should be selected on the basis of their size (lesions with the longest diameter) and their suitability for accurate repeated measurements (either by imaging techniques or clinically).
- A sum of the longest diameter (LD) for *all target lesions* will be calculated and reported as the baseline sum LD. The baseline sum LD will be used as reference by which to characterize the objective tumor.

All other lesions (or sites of disease) should be identified as ***non-target lesions*** and should also be recorded at baseline. Measurements of these lesions are not required, but the presence or absence of each should be noted throughout follow-up.

Response Criteria

Evaluation of target lesions

Complete Response (CR):	Disappearance of all target lesions
Partial Response (PR):	At least a 30% decrease in the sum of the LD of target lesions, taking as reference the baseline sum LD
Progressive Disease(PD):	At least a 20% increase in the sum of the LD of target lesions, taking as reference the smallest sum LD recorded since the treatment started or the appearance of one or more new lesions
Stable Disease (SD):	Neither sufficient shrinkage to qualify for PR nor sufficient increase to qualify for PD, taking as reference the smallest sum LD since the treatment started

Evaluation of non-target lesions

Complete Response (CR):	Disappearance of all non-target lesions and normalization of tumor marker level
Incomplete Response/ Stable Disease (SD):	Persistence of one or more non-target lesion(s) or/and maintenance of tumor marker level above the normal limits
Progressive Disease (PD):	Appearance of one or more new lesions and/or unequivocal progression of existing non-target lesions (1)

(1) Although a clear progression of “non target” lesions only is exceptional, in such circumstances, the opinion of the treating physician should prevail and the progression status should be confirmed later on by the review panel (or study chair).

Evaluation of best overall response

The best overall response is the best response recorded from the start of the treatment until disease progression/recurrence (taking as reference for PD the smallest measurements recorded since the treatment started). In general, the patient's best response assignment will depend on the achievement of both measurement and confirmation criteria

Target lesions	Non-Target lesions	New Lesions	Overall response
CR	CR	No	CR
CR	Incomplete response/SD	No	PR
PR	Non-PD	No	PR
SD	Non-PD	No	SD
PD	Any	Yes or No	PD
Any	PD	Yes or No	PD
Any	Any	Yes	PD

- Patients with a global deterioration of health status requiring discontinuation of treatment without objective evidence of disease progression at that time should be classified as having “symptomatic deterioration”. Every effort should be made to document the objective progression even after discontinuation of treatment.
- In some circumstances it may be difficult to distinguish residual disease from normal tissue. When the evaluation of complete response depends on this determination, it is recommended that the residual lesion be investigated (fine needle aspirate/biopsy) to confirm the complete response status.

Confirmation

- The main goal of confirmation of objective response is to avoid overestimating the response rate observed. In cases where confirmation of response is not feasible, it should be made clear when reporting the outcome of such studies that the responses are not confirmed.
- To be assigned a status of PR or CR, changes in tumor measurements must be confirmed by repeat assessments that should be performed no less than 4 weeks after the criteria for response are first met. Longer intervals as determined by the study protocol may also be appropriate.
- In the case of SD, follow-up measurements must have met the SD criteria at least once after study entry at a minimum interval (in general, not less than 6 weeks) that is defined in the study protocol

Duration of overall response

- The duration of overall response is measured from the time measurement criteria are met for CR or PR (whichever status is recorded first) until the first date that recurrence or PD is objectively documented, taking as reference for PD the smallest measurements recorded since the treatment started.

Duration of stable disease

- SD is measured from the start of the treatment until the criteria for disease progression are met, taking as reference the smallest measurements recorded since the treatment started.
- The clinical relevance of the duration of SD varies for different tumor types and grades. Therefore, it is highly recommended that the protocol specify the minimal time interval required between two measurements for determination of SD. This time interval should take into account the expected clinical benefit that such a status may bring to the population under study.

Response review

- For trials where the response rate is the primary endpoint it is strongly recommended that all responses be reviewed by an expert(s) independent of the study at the study's completion. Simultaneous review of the patients' files and radiological images is the best approach.

Reporting of results

- All patients included in the study must be assessed for response to treatment, even if there are major protocol treatment deviations or if they are ineligible. Each patient will be assigned one of the following categories: 1) complete response, 2) partial response, 3) stable disease, 4) progressive disease, 5) early death from malignant disease, 6) early death from toxicity, 7) early death because of other cause, or 9) unknown (not assessable, insufficient data).
- All of the patients who met the eligibility criteria should be included in the main analysis of the response rate. Patients in response categories 4-9 should be considered as failing to respond to treatment (disease progression). Thus, an incorrect treatment schedule or drug administration does not result in exclusion from the analysis of the response rate. Precise definitions for categories 4-9 will be protocol specific.
- All conclusions should be based on all eligible patients.
- Subanalyses may then be performed on the basis of a subset of patients, excluding those for whom major protocol deviations have been identified (e.g., early death due to other reasons, early discontinuation of treatment, major protocol violations, etc.). However, these subanalyses may not serve as the basis for drawing conclusions concerning treatment

efficacy, and the reasons for excluding patients from the analysis should be clearly reported.

- The 95% confidence intervals should be provided.

Procedures for PET examination

12.3.1.1 Patient preparation

For FDG-PET and FLT-PET patients must refrain from ingestion for 12 hours prior to the PET examination; only the ingestion of carbohydrate-free drinks is permitted. The current glucose level should be measured before starting FDG-PET. Fasting glucose level over 130 mg/dl or known diabetes mellitus are exclusion criteria for FDG-PET. The physicians may optionally administer regular insulin (e.g. 2 units) if blood glucose levels are unexpectedly high, provided that monitoring and emergency care are assured. For FLT-PET no special preparation is necessary.

12.3.1.2 Administration of tracers, scanning and data evaluation

18F-FDG and 18F-FLT will be synthesized at the Max-Planck-Institute for Neurological Research at the University of Cologne following standard procedures. A dose of 370 MBq FDG and 300 MBq FLT will be injected intravenously for whole-body examination by PET. Scanning will commence ~45-60 minutes after injection of tracers. PET instruments with full-ring detectors will be applied. Emission data will be corrected for attenuation, scatter and random and will be reconstructed to plains of 3.125 mm width and 128x128 voxels. For quantification of tracer uptake, standard methods, such as standardized uptake values (SUV) will be applied.

Procedures for EGFR and KRAS gene sequencing

12.3.1.3 Sample procurement

Samples will be obtained from the same tumor biopsy specimen used for histological diagnosis. The primary pathologist decides which part of a sample can be used for biological analyses. All sample procurement and storage processes have already been established at MTBTI, Dept I of Internal Medicine, University Clinic Cologne and are highly standardized as standard operating procedures (SOPs).

12.3.1.4 Estimation of tumor cell purity and preparation of genomic DNA

The primary pathologist will estimate the tumor cell purity at the margin of dissection where the snap-frozen sample has been cut off. When the tumor cell population is estimated to be >70%, DNA will be directly prepared from 20 µm sections prepared using standard section techniques. DNA will be prepared using the Puregene™ kit (Gentra), following the recommendations of the manufacturer. If the tumor purity is estimated to be <70%, tumor cells

will be enriched using a laser-pressure catapulting microdissection instrument (PALM Microlaser Technologies). DNA will be purified from these samples using the Puregene™ kit.

12.3.1.5 Amplification and direct sequencing of the EGFR and KRAS

Pre-treatment biopsy material will be analyzed for EGFR mutational status by exon re-sequencing. Exon-spanning primers will be designed using Primer3 software in the introns adjacent to exons and target sequences will be amplified by single-round PCR. Purified PCR products will be bi-directionally sequenced (sequences controlled for bi-allelic target amplification; quality threshold: Phred score >30).

12.3.1.6 Sequence analyses

The resulting electropherograms will be manually and computationally (software: DNAsis) compared to germline sequences of the EGFR gene and KRAS gene (GeneID: 1956; RefSeq: NM_005228, GeneID 3845, RefSeq: NM_033360). Splice sites will also be analyzed. Sequence variations will be classified mutations if they are present in both bi-directional target electrophergrams and if they do not correspond to known germline polymorphisms. Furthermore, sequencing of control DNA from non-tumoral tissue (peripheral blood) is mandatory in those cases with a mutation to control for rare germline polymorphisms.

Quality control

Quality control is according to the recommendations for tumor depiction with FDG of the German Society for Nuclear Medicine (Knapp 1999).

Procedure for the performance of computed tomography

To optimize the assessment of tumor tissue volume using computed tomography, the following technical criteria should be aimed at:

- Spiral CT technique
- Intravenous contrast medium administered for all examinations
- Oral contrast medium for abdominal CT
- Spiral CT neck: (collimation/table shift/increment) 5/6/4
- Spiral CT chest: 5/8/4
- Spiral CT abdomen: 8/10/8

Computed tomography is inadequate:

- with layer thickness > 1 cm and table shift > 1.5 cm

- without or with too weak intravenous contrasting, which fails to differentiate blood vessels from neighboring tumor and tumor-affected lymphatic tissue reliably
- without or with too weak oral contrasting, which fails to differentiate intestine from neighboring lymphoma tissue reliably.

12.4 Patient's consent

Guide to patient briefing

Patient briefing is to contain all the points 1. to 6. below. A witness is required only if the patient is illiterate.

1. The trial

Short explanation of the trial title, verdict of the ethical committee, recruitment, insurance, aim, experimental procedure, procedure and advantages of the trial, transfer of data and test material, freedom of decision, absence of additional trial-related costs.

2. Nature of the disease

Short description of symptoms, clinical matters, response to treatment and prognosis as well as age distribution and the prognosis of untreated disease.

3. Protocol

A. *Target group*:
NSCLC patients in stage IIIB/IV

B. *Treatment*:
Therapy with erlotinib (Tarceva®) until week 6
In the course of the study patients must undergo repeated PET examinations to evaluate response to therapy.

C. *Aim:*
To evaluate the accuracy of FDG-/FLT-PET analyses for early prediction of erlotinib non-progression in patients with NSCLC

D. *Effects*:
Lessening of symptoms such as weight loss, loss of appetite, cough, hemoptysis, dyspnea, chronical pneumonia; improvement of general wellbeing.

E. *Possible side effects:*

Mainly mild diarrhea and rashes were observed. In 0.6% of the patients interstitial lung disease was observed.

F. *Approval:*
Erlotinib is approved in the EU (including Germany), Switzerland and the US for the treatment of non-small cell lung cancer after failure of at least one prior chemotherapy regimen. Erlotinib so far is not approved for first line treatment of lung cancer. In phase II trials, however, erlotinib has shown therapeutic activity in patients with NSCLC treated in the first line situation comparable to chemotherapy. Tarceva is the only drug in the EGFR inhibitor class to demonstrate an increase in overall survival in a phase III clinical trial in advanced NSCLC patients. There is thus experience with the safety issues connected with this drug. Importantly, the adverse events reported for erlotinib are mild as compared to those of chemotherapy. Moreover, erlotinib has been reported to be at least as effective as chemotherapy in the treatment of relapsed lung cancer.

G. *PET:*
PET will be performed for identification of an early response to treatment with erlotinib. Both tracers (FDG and FLT) are well tolerated, allergic reactions are only very rarely observed. FLT is not approved for PET analyses in lung cancer. However, FLT has been successfully administered to more than 150 patients worldwide, up to now no adverse event has been reported. Moreover, at the Max-Planck Institute for Neurological Research at the University of Cologne more than 40 patients have been investigated using FLT-PET and no single adverse event has been noted, up to now. Additionally, this indicates an extensive experience with the synthesis and administration of FLT in humans. Beyond the radioactivity issues connected with FLT, this tracer can thus be considered safe.

5. Follow-up examinations

It is explained to the patient that regular monitoring examinations over a time-span till the follow up period end date are required in his/her own interest and especially in the interest of future patients. The results of these examinations will be reported to the trials coordination center.
The patient is told that he/she can be contacted by the trials coordination center, if he/she has agreed thereto.

6. Patients freedom of decision

The patient can reconsider his decision to participate in the trial at any time. If the patient refuses participation, he/she is free to choose further treatment.

7. Further procedures before patient´s consent.

After the patient´s information the patient will be asked for his/her queries and he/she will have at least 24 h in addition to consider his/her participation in the trial. The written informed consent of the patient has to be obtained before any study-related activities are carried out. It

must be signed and personally dated by the patient and by the investigator/person designated by the investigator to conduct the informed consent discussion. To avoid any misunderstandings, the informed consent should be signed by the patient in the presence of the investigator/person designated by the investigator to conduct the informed consent discussion. The signed and dated declarations of informed consent will remain at the investigator´s site and must be safely archived by the investigator so that the forms can be retrieved at any time for monitoring, auditing and inspection purposes. A copy of the signed and dated information and consent should be provided to the patient prior to participation.

Patienteninformation und Einverständniserklärung

ERLOPET

Titel der Studie:

Eine klinische Studie zur Evaluation von FDG-/FLT-PET zur frühen Vorhersage des Nichtansprechens von Patienten mit der Erstdiagnose eines fortgeschrittenen nicht-kleinzelligen Bronchialkarzinoms auf die Behandlung mit Erlotinib (Tarceva®) und zum Zusammenhang von PET-Befunden und molekularen Markern.

Sehr geehrte Patientin, sehr geehrter Patient,

wir möchten Sie um Ihr Einverständnis zur Teilnahme an einer klinischen Studie bitten. Die folgenden Seiten sollen Sie eingehend über die geplante Studie informieren und Ihnen helfen, die Entscheidung über Ihre Teilnahme zu treffen.

Ihr behandelnder Arzt hat Sie darüber aufgeklärt, dass Sie an einem nicht-kleinzelligen Bronchialkarzinom erkrankt sind, einer Krebserkrankung der Lunge.

In der hier vorliegenden wissenschaftlichen Studie wollen die in der Lungenkrebs Studiengruppe am Kölner Universitätsklinikum (LUNG CANCER GROUP COLOGNE; LCGC) zusammengeschlossenen Ärzte einen neuen Behandlungsansatz testen, der eine Verbesserung der derzeitigen Therapie zum Ziel hat.

Das Ziel dieser Studie besteht darin, die geeigneten Patienten für eine Behandlung mit dem neuen Krebsmittel Erlotinib (Tarceva®) schon sehr frühzeitig nach Beginn der Therapie zu identifizieren. Erlotinib ist ein neues Medikament, das anders als klassische Chemotherapeutika wirkt. Erlotinib hemmt einen Wachstumsrezeptor auf den Krebszellen und blockiert auf diese Weise selektiv Eiweisstoffe, die das unkontrollierte Wachstum der Krebszellen aufrechterhalten. In bereits durchgeführten Therapiestudien konnte verläßlich nachgewiesen werden, daß diese im Vergleich zur Chemotherapie gezieltere Therapie wirksam ist, mit deutlich geringeren Nebenwirkungen.

Da mehrere Medikamente für die Therapie des Bronchialkarzinoms zur Verfügung stehen, ist eine frühe Identifizierung derjenigen Patienten wünschenswert, die nicht auf die Therapie mit Erlotinib ansprechen. Diesen Patienten könnte dann sehr schnell eine andere wirksame Therapie gegeben werden. Deshalb soll in dieser Studie geprüft werden, inwieweit die bildgebende Untersuchungsmethode PET (= Positronen-Emissions-Tomographie) in der Lage ist, frühzeitig diejenigen Patienten zu identifizieren, bei denen eine Therapie mit Erlotinib erfolglos ist. Insbesondere soll geprüft werden, ob das neuartige Strahlendiagnostikum 18-Fluorothymidin (FLT), das besonders schnell Veränderungen im Tumorwachstum aufdecken kann, hierzu in der Lage ist.

Zusätzlich zu den PET Untersuchungen sind genetische Untersuchungen des Tumorgewebes vorgesehen, das zur Diagnosesicherung der Erkrankung entnommen wurde. Bei dieser Untersuchung wird geprüft, ob der Ihnen entnommene Tumor bestimmte genetische Eigenschaften aufweist, von denen bekannt ist, daß sie einen starken Einfluß auf den Therapieerfolg von Erlotinib haben. Das Ziel der vorliegenden Studie ist es, die Zusammenhänge zwischen diesen genetischen Markern, der PET-Untersuchung und Ihrem Therapieerfolg zu klären. Auf diese Weise soll es in Zukunft ermöglicht werden, frühzeitig diejenigen Patienten zu identifizieren, die von einer Therapie mit Erlotinib nicht profitieren, damit diese dann mit einer anderen Therapie behandelt werden können.

Erlotinib ist in der Europäischen Union (also auch in Deutschland) bereits für die Behandlung des nicht-kleinzelligen Bronchialkarzinoms nach dem Versagen von mindestens 1 Standardchemotherapie zugelassen, nicht aber als Erstbehandlung. Der Einsatz dieses Medikamentes als erste Therapie bei nicht-kleinzelligen Bonchialkarzinom wird derzeit in mehreren Studien überprüft. Über 100 Patienten mit Bronchialkarzinom wurden schon mit Erlotinib als erster Therapie im Rahmen von Studien behandelt und die Ergebnisse sind sehr vielversprechend, d.h. die Ansprechraten liegen im Bereich der zurzeit wirksamsten Chemotherapien. Die genaue Beschreibung dieses neuen Medikamentes, seiner möglichen Nebenwirkungen sowie die Beschreibung von PET- und Labor-Untersuchungen finden Sie in dieser Information in den folgenden Abschnitten.

Ablauf der Behandlung

Erlotinib (Tarceva®) wird Ihnen als Tabletten verabreicht, die Sie täglich (am besten 1 Stunde nach einer Mahlzeit) einnehmen sollen. Sie nehmen jeweils eine Tablette pro Tag ein, und Sie sollten 2 Stunden nach der Einnahme nicht essen.

Ob diese Behandlung bei Ihnen erfolgreich ist, soll mittels verschiedener bildgebender Verfahren geklärt werden:

- Computertomographie (CT) mit Röntgenkontrastmittel, ein röntgenologisches Standardverfahren,
- Positronen-Emissions-Tomographie (PET) mit Fluor-18 Fluorodeoxyglukose (FDG), ein diagnostisches nuklearmedizinisches Standardverfahren mit einem radioaktiven Arzneimittel,
- Positronen-Emissions-Tomographie (PET) mit Fluor-18 Fluorothymidin (FLT), ebenfalls ein diagnostisches nuklearmedizinisches Verfahren mit einem radioaktiven Arzneimittel.

Alle drei Untersuchungsverfahren sollen vor Beginn der Behandlung mit Erlotinib (Tarceva®), eine Woche nach Beginn dieser Behandlung und wiederum sieben Wochen später zum Ende des Behandlungszyklus zum Einsatz kommen. Danach wird entschieden, ob eine Fortführung der Behandlung mit Erlotinib erfolgsversprechend ist.

Bei gutem Ansprechen nehmen Sie die Erlotinib-Tabletten nach den 6 Wochen weiter ein, solange die Wirkung anhält. Bei unzureichendem Ansprechen werden Sie, wenn Sie einverstanden sind und es Ihr Gesundheitszustand zulässt, mit einer Chemotherapie

weiterbehandelt. Die Chemotherapie wird Ihnen entweder als Infusion von mehreren Substanzen (kombinierte Chemotherapie) oder von einer einzelnen Substanz (Monotherapie) zu genau festgelegten Zeiten verabreicht. Für die Chemotherapie ist, je nach Wahl des Medikaments, eine eigene, ausführliche Aufklärung vorgesehen.

Meldung

Jeder Patient, der sich für die Teilnahme an der Studie entscheidet und seine schriftliche Einwilligung abgibt, wird in die Studie gemeldet.

Untersuchungen im Rahmen der Studie

Im Rahmen dieser Studie wird eine Reihe von Untersuchungen durchgeführt mit dem Ziel, den körperlichen Zustand des Patienten vor, während und nach der Behandlung so gut wie möglich zu erfassen. Ohne diese Untersuchungen kann ein möglicher Nutzen der Behandlung nicht hinreichend festgestellt werden. Die Gewebeproben von der für die Erstellung der Diagnose entnommenen Probe werden zum Zwecke wissenschaftlicher Untersuchungen im Labor der LCGC-Gruppe untersucht.

Untersuchungen vor der Therapie

Vor der Behandlung erfolgen die **Erhebung der Krankengeschichte** sowie die **körperliche Untersuchung** durch den behandelnden Arzt. Sollten Sie regelmäßig Medikamente einnehmen, teilen Sie dieses Ihrem Arzt bitte mit.
Eine umfangreiche Labordiagnostik (d.h. Blutentnahme) sowie eine Testung auf mögliche Viruserkrankungen wie Hepatitis und HIV (Humanes Immundefizienz-Virus) wird vorgenommen.
Hepatitiden B und C sind Leberentzündungen verursacht durch die Hepatitis B-, bzw. C-Viren und entstehen vor allem durch Blutprodukte, kontaminierte Instrumente, und sexuelle Kontakte mit einer infizierten Person. Hepatitis B ist auch von der Mutter auf das Kind übertragbar. Die beiden Hepatitiden können chronisch verlaufen und stellen eine zusätzliche Belastung für die Leber dar. Sollte bei Ihnen neu eine solche Hepatitis diagnostiziert werden, so würde, wie vom Gesetzgeber vorgeschrieben, eine namentliche Meldung an das Gesundheitsamt erfolgen.
Das erworbene Immunschwächesyndrom (AIDS) ist eine lebensbedrohliche Viruserkrankung. Der Erreger dieser Krankheit (Human Immune Deficiency Virus = HIV) schädigt das körpereigene Abwehrsystem. Er wird vor allem durch den direkten Kontakt mit Blut und Körpersekreten und beim Geschlechtsverkehr übertragen. Die Krankheit selbst bricht in der Regel erst 5-10 Jahre nach der Infektion aus. Im Rahmen einer ausgebrochenen AIDS-Erkrankung kommt es zu einer Vielzahl begleitender, weiterer Erkrankungen: Infektion mit Bakterien, Viren, Pilzen und Parasiten, Entstehung von Tumoren des Lymph- und Gefäßsystems (Kaposi-Sarkom) und Ausfälle des Nervensystems. In Ihrem eigenen Interesse sollten Sie anlässlich dieser Blutabnahme die Gelegenheit nutzen und einen HIV-Test durchführen lassen. Das Wissen über eine HIV-Infektion dient Ihnen und auch dem Schutz Ihres Partners. Es ist verständlich, wenn Sie sich scheuen, sich einem solchem Test zu unterziehen, denn die Mitteilung über ein positives Testergebnis bedeutet eine schwere Belastung für Sie. Sie können sicher sein, dass ein positives Ergebnis eines solchen Tests nicht weitergegeben wird, denn derartige Tests unterliegen der strengsten ärztlichen

Schweigepflicht. Es erfolgt allerdings, auch hier wie vom Gesetzgeber vorgeschrieben, eine anonyme Meldung an die zuständige Behörde, in diesem Fall das Robert-Koch-Institut. Darüber hinaus existieren wirksame Therapien gegen eine HIV Infektion. Ohne Ihr Einverständnis zu Untersuchungen auf Hepatitis-B, -C und HIV-Infektion kann keine Behandlung innerhalb dieser Studie erfolgen.

Das neue Medikament Erlotinib wirkt in der Krebszelle auf eine ganz bestimmte Stelle in einem Molekül an der Oberfläche der Krebszelle, dem so genannten EGF- (Epidermal Growth Factor) Rezeptor. Kürzlich wurde nachgewiesen, dass bestimmte Veränderungen in diesem Molekül (genetische Mutationen) zu einem besseren Therapie-Ansprechen bei einer Therapie mit Erlotinib führen, bestimmte Änderungen in einem anderen Molekül zu einem schlechteren Ansprechen. Aus diesem Grund wird die Probe, die Ihnen zur Diagnose Ihrer Erkrankung entnommen wurde, untersucht. Nach Ihrer Einwilligung in die Studie wird der Pathologe aufgefordert, uns die Probe zuzusenden, die Ihnen zur Erstellung der Diagnose entnommen wurde.

Ferner ist die Überprüfung einiger weiteren Organfunktionen vor Beginn der Therapie erforderlich. Aufgrund dieser Voruntersuchungen können mögliche Nebenwirkungen der Therapie gezielter festgestellt werden. Zu diesen Untersuchungen gehören: **EKG** (Elektrokardiogramm), **Echokardiogramm** (Ultraschalluntersuchung des Herzens) und die **Lungenfunktionsprüfung.**

Zur exakten Beschreibung der Krankheitsausdehnung werden verschiedene bildgebende Untersuchungsverfahren eingesetzt: **Computertomographien** und **FDG- und FLT-PET** Untersuchungen. In Einzelfällen kann auch eine **Kernspintomographie** nötig sein.

Warum sollen mehrere bildgebende Untersuchungsverfahren bei Ihnen angewendet werden?

Die Computertomographie bildet die Form und Größe des Lungentumors in optimaler Weise ab. Das radioaktive Medikament FDG und der radioaktive Stoff FLT stellen demgegenüber Stoffwechselvorgänge dar, FDG (Fluorodeoxyglukose) den Zuckerstoffwechsel und FLT (Fluorothymidin) das Zellwachstum. In anderen Studien haben sich deutliche Hinweise ergeben, dass unter Anwendung der PET mit Fluorodeoxyglukose und mit Fluorothymidin ein Therapieansprechen frühzeitiger zu erkennen ist als mit der Computertomographie. So ist die Abnahme des erhöhten Zuckerstoffwechsels oder die Abnahme der Zellvermehrung auch bei unveränderter Tumorgröße ein klares Zeichen für ein Therapieansprechen. Dementsprechend kann in diesen frühen Stadien ein Ansprechen nur mittels PET, jedoch nicht mittels CT dargestellt werden.
Darüber hinaus wird die PET mit FDG und FLT als Ganzkörper-Untersuchung durchgeführt, es werden also der Schädel, die Halsweichteile, der Brustkorb, der Bauchraum und das Becken untersucht. Sollten sich hierbei anderweitige Tumorherde (Metastasen) zeigen, ist unter Umständen eine zusätzliche Behandlungsmaßnahme empfehlenswert.

Welche Vorteile hat die PET Untersuchung mit Fluorodeoxyglukose und mit Fluorothymidin für Sie?

Es kann eine Aussage getroffen werden, ob die Behandlung des Bronchialkarzinoms mit Erlotinib (Tarceva®) bei Ihnen wirksam ist. Ihr behandelnder Arzt erhält außerdem Informationen, ob sich in Ihrem Körper weitere Krankheitsherde gebildet haben, die ggf. einer gesonderten zusätzlichen Behandlung bedürfen.

Nach Abschluss der vollständigen diagnostischen Untersuchungen, die etwa eine Woche beanspruchen, kann zügig mit der Therapie begonnen werden.

Durchführung der PET-Untersuchungen

Die radioaktiv markierten Diagnostika Fluorodeoxyglukose und Fluorothymidin werden jeweils in eine Armvene eingespritzt. Die PET-Untersuchung bedeutet für Sie jeweils einen Zeitaufwand von 2 bis 3 Stunden, wobei die Aufnahmezeit im Untersuchungsgerät etwa 1 Stunde beträgt. Am Untersuchungstag dürfen Sie bis zur Durchführung der PET-Untersuchung nichts essen. Erlaubt sind aber die Einnahme ihrer Medikamente und das Trinken von Wasser, schwarzem Kaffee **ohne** Zucker oder von Tee **ohne** Zucker. Patienten mit einem Diabetes mellitus oder mit erhöhtem Blutzucker reichern das radioaktive Medikament FDG in geringerer Intensität an und werden nicht im Rahmen dieser Studie untersucht.

Durchführung der Erlotinib-Therapie

Ab dem ersten Tag der Behandlung nehmen Sie bitte eine Tablette Erlotinib (Tarceva®, 150mg) ein. Es wird empfohlen, die Tablette 1 Stunde nach dem Essen einzunehmen und anschließend 2 Stunden nicht zu essen.

Untersuchungen während der Erlotinib (Tarceva®) Therapie

Während der Erlotinib-Therapie wird Ihr Arzt Sie in regelmäßigen Abständen zur Kontrolle Ihrer gesundheitlichen Verfassung, ihrer Blutwerte und unter Umständen zur Durchführung von Röntgen- oder Ultraschalluntersuchungen einbestellen. Die Häufigkeit dieser Untersuchungen hängt von Ihrem Befinden und der Verträglichkeit der Therapie ab.
Eine Woche nach Beginn der Therapie erfolgen eine FDG-PET- und eine FLT-PET-Untersuchung. Diese Untersuchungen dienen der frühen Feststellung eines Ansprechens.

Überprüfung des Behandlungserfolges (Restaging)

Eine Überprüfung des Therapieerfolges wird nach der 6-wöchigen Therapie mit Erlotinib (Tarceva®) durchgeführt. Bei dieser Kontrolle werden erneut eine körperliche Untersuchung, Blutuntersuchungen, CT- und FDG- und FLT-PET Untersuchungen durchgeführt.
Bei dieser Untersuchung wird festgelegt, ob bei Ihnen die Behandlung mit Erlotinib (Tarceva®) weiter fortgeführt wird (bei gutem Ansprechen), oder ob Sie mit einer anderen Chemotherapie (bei unzureichendem Ansprechen) behandelt werden. Falls Sie sich zusammen mit Ihrem betreuenden Arzt für die Durchführung einer Chemotherapie entscheiden, werden Sie über die Wahl des Zytostatikums, die Erfolgsaussichten und die Nebenwirkungen gesondert ausführlich aufgeklärt.

Nebenwirkungen/Risiken der FDG- und FLT-PET Untersuchung

Die beiden radioaktiven Diagnostika werden im Allgemeinen sehr gut vertragen, da es sich schlicht um radioaktiv markierten Zucker (FDG) bzw. um einen radioaktiv markierten und veränderten Baustein der DNS (Träger der Erbinformation) handelt (FLT). Allergische Reaktionen sind sehr selten.

Die PET-Untersuchungen sind mit einer geringen Strahlenexposition verbunden, die etwa in der Größenordnung einer CT-Untersuchung liegt. Deshalb werden Frauen im gebärfähigen Alter bei einer vermuteten oder bestehenden Schwangerschaft und während der Stillzeit nicht mittels PET untersucht.

Nebenwirkungen/Risiken der Erlotinib (Tarceva®)-Therapie

Die mit der Erlotinib-(Tarceva®) verbundenen Nebenwirkungen sind erfahrungsgemäß nur von geringer bis mittlerer Schwere. Eine häufige unerwünschte Nebenwirkung ist das Auftreten eines Hautausschlags. Dieser Ausschlag ähnelt oft einer Akne, tritt zumeist nach einigen Tagen Therapie auf, verläuft aber in den meisten Fällen mild bis moderat. Schwere Fälle, die auch zum Abbruch der Therapie führen können, sind selten (< 10%). Auch Durchfall tritt häufig auf, allerdings in den meisten Fällen in milder Form. Weiter wird über Müdigkeit, Appetitlosigkeit, Husten, Infektionen, Übelkeit, Erbrechen und Bauchschmerzen berichtet. Selten konnte eine Erhöhung der Leberwerte ohne Krankheitssymptome festgestellt werden. Sehr selten entwickelten Patienten eine Lungenerkrankung, eine sogenannte interstitielle Lungenentzündung. Eine detaillierte Aufklärung über Erlotinib finden Sie im Anhang zu dieser Patienteninformation.

Ausgeschlossene Teilnahme an anderen Forschungsprojekten vor und nach dem Ablauf dieser klinischen Prüfung

Während der gesamten Zeit, in der Sie an dieser Studie teilnehmen, werden Sie im Universitätsklinikum Köln ambulant (und falls notwendig stationär) von den für die Studie zuständigen Ärzten behandelt. Dadurch wird verhindert, dass Sie gleichzeitig an anderen klinischen Prüfungen oder Forschungsprojekten teilnehmen oder vor Ablauf einer erforderlichen Karenzzeit an dieser klinischen Prüfung teilnehmen. Die gleichzeitige Teilnahme an mehreren klinischen Studien oder Forschungsprojekten kann in einem ungünstigen Fall zur Häufung von unerwünschten Ereignissen und Nebenwirkungen führen und kann die Aussagekraft der Fragestellung dieser Studie beeinflussen.

Andere Behandlungsmöglichkeiten

Falls Sie sich entscheiden, nicht an dieser Studie teilzunehmen, wird Ihr Arzt Ihnen erklären, welche anderen Behandlungsmöglichkeiten Ihnen zur Verfügung stehen.

Weitere Informationen

Dieses Studienprotokoll wurde der Ethikkommission der Universität zu Köln zur Begutachtung vorgelegt und positiv bewertet. Die Teilnahme an dieser klinischen Studie ist selbstverständlich freiwillig. Ihr Arzt wird Sie bitten, eine Einverständniserklärung zu unterzeichnen und damit auch zu bestätigen, dass Sie vollständig über die Studie informiert wurden und deren Zielsetzung verstehen.

Sie haben jedoch das Recht, Ihre Einwilligung zur Teilnahme an der Studie jederzeit und ohne Angabe von Gründen zu widerrufen, ohne dass das Vertrauensverhältnis zu Ihrem behandelnden Arzt darunter leidet. Aus Sicherheitsgründen sollten jedoch bei vorzeitigem Studienabbruch abschließende Untersuchungen sowie Nachsorgeuntersuchungen stattfinden. Bei vorzeitigem Abbruch werden Sie außerhalb dieser Studie nach bestem medizinischen Wissen weiter behandelt.
Sollte Ihr behandelnder Arzt der Meinung sein, dass eine weitere Teilnahme an dieser Studie für Sie von Nachteil ist, so kann er Ihre Behandlung im Rahmen dieser Studie auch ohne Ihre Zustimmung beenden. In diesem Fall würden Sie außerhalb dieser Studie nach bestem medizinischen Wissen weiter behandelt. Sie werden selbstverständlich auch über alle neuen Informationen, die für die Teilnahme an der Studie relevant sein könnten, umgehend informiert.

Der Nutzen Ihrer Teilnahme besteht in der Möglichkeit, durch eine neue Therapie erfolgreich behandelt zu werden. Mit den Ergebnissen dieser Studie lassen sich möglicherweise wertvolle Erkenntnisse für die zukünftige Behandlung Ihrer Erkrankung erzielen. Es kann Ihnen jedoch nicht garantiert werden, dass Sie durch eine Teilnahme an dieser Studie einen Vorteil haben werden.

Da die in dieser Studie eingesetzten Substanzen möglicherweise das ungeborene Leben schädigen können, sind Schwangere von einer Teilnahme ausgeschlossen. Frauen im gebärfähigen Alter müssen durch sichere Verhütungsmethoden gewährleisten, dass eine Schwangerschaft für die Dauer der Behandlung ausgeschlossen ist. Auch über einen Zeitraum von einem Jahr nach der Behandlung empfehlen wir, eine Schwangerschaft zu vermeiden.

Wichtiger Hinweis

Vor der Teilnahme an dieser Studie ist es wichtig, Sie auf Folgendes hinzuweisen:

1. Das Medikament Erlotinib (Tarceva®) ist in Deutschland für die Ersttherapie des nicht-kleinzelligen Bronchialkarzinoms nicht zugelassen. Es ist in Europa und den USA bereits zugelassen für die Behandlung des nicht-kleinzelligen Bronchialkarzinoms nach dem Versagen von mindestens 1 Standard-Chemotherapie. Mittlerweile wurden weltweit tausende Patienten mit Erlotinib behandelt. Deshalb besteht eine entsprechend große Erfahrung mit den im Vergleich zur Chemotherapie geringen Nebenwirkungen dieses Medikamentes.
2. Das radioaktive Diagnostikum Fluorothymidin ist in Deutschland kein arzneimittelrechtlich anerkanntes Medikament. Mittlerweile sind insgesamt über 250 Patienten mit FLT untersucht worden und es konnten bislang keine unerwünschten

Wirkungen nachgewiesen werden. Die Strahlenwirkungen sind identisch zu jenen von FDG.

Patientenversicherung

Gemäß den Vorschriften des §40 Abs. 1 Nr. 8 und Abs. 3 des deutschen Arzneimittelgesetzes (AMG) besteht für Sie eine Patientenversicherung (Vers. Nr.70-005621716-3) bei dem Gerling-Konzern Düsseldorf (Ansprechpartner: Bernhard Hoppe).
Es liegt eine Versicherung vor, sowohl für den 1-jährigen Zeitraum der Aufnahme von Patienten in die Studie, als auch für die Dauer von 1 Jahr nach Meldung in die Studie (Nachbeobachtungszeitraum) für jedes Ereignis in Zusammenhang mit der Behandlung in der Studie.

Somit besteht für jeden einzelnen Patienten eine Versicherung von 1 Jahr nach Meldung in die Studie.

Zusätzlich besteht ein Versicherungsschutz nach dem Atomgesetz. Dieser greift bei jedwedem Schaden in Zusammenhang mit der Strahlenbelastung im Rahmen der Studie. Dieser Versicherungschutz gilt für 30 Jahre nach Einschluß in die Studie. Innerhalb der ersten 10 Jahre erfolgt der Versicherungsschutz durch den Gerlingkonzern. Für den folgenden Zeitraum liegt eine Bürgschaft des Landes NRW für eventuelle Gesundheitsschäden vor.

Der Versicherungsschutz umfasst Gesundheitsschäden, die im zeitlichen und ursächlichen Zusammenhang mit dieser klinischen Prüfung aufgetreten sind. Die maximale Deckungssumme pro versicherter Person beträgt 500.000 €. Die Höchstleistung für alle Versicherungsfälle innerhalb dieser Studie beträgt 50.000.000 €.

Der Versicherungsschutz ist insbesondere an folgende Bedingungen geknüpft:

1. Während der Dauer der klinischen Prüfung dürfen Sie sich einer anderen medizinischen Behandlung nur nach Rücksprache mit Ihrem behandelnden Arzt unterziehen. Dies gilt nicht in einem medizinischen Notfall; Ihr behandelnder Arzt ist jedoch von der Notfallbehandlung unverzüglich zu unterrichten.
2. Eine Gesundheitsschädigung, die als Folge der klinischen Prüfung eingetreten sein könnte, ist dem Versicherer unverzüglich zu melden.
3. Im möglichen Schadensfall sind von Ihnen alle zweckmäßigen Maßnahmen zu treffen, die der Aufklärung der Ursache und des Umfangs des eingetretenen Schadens und der Minderung dieses Schadens dienen.
4. Auf Verlangen des Versicherers ist der behandelnde Arzt – als solcher gilt auch ein Konsiliararzt oder ein gutachterlich tätiger Arzt – zu veranlassen, einen Bericht über die Gesundheitsschädigung und, nach Abschluss der ärztlichen Behandlung, einen Schlussbericht zu erstatten, außerdem ist Sorge zu tragen, dass alle von dem Versicherer geforderten Berichte des behandelnden Arztes geliefert werden. Alternativ können Sie Ihren behandelnden Arzt von der ärztlichen Schweigepflicht entbinden, damit der Versicherer die vorab genannten Berichte direkt beim Arzt anfordern kann.

5. Die behandelnden Ärzte - auch diejenigen, von denen Sie aus anderen Anlässen behandelt oder untersucht worden sind – und die Sozialversicherungsträger sowie andere Versicherer – wenn dort die Gesundheitsschädigung gemeldet ist - sind zu ermächtigen, dem Versicherer auf Verlangen Auskunft zu erteilen.
6. Hat der Versicherungsfall den Tod zur Folge, so ist dies unverzüglich anzuzeigen, und zwar auch dann, wenn eine Meldung nach Abs. 2 bereits erfolgt ist. Dem Versicherer ist das Recht zu verschaffen, eine Obduktion durch einen von ihm beauftragten Arzt vornehmen zu lassen.

Die Meldungen sind unter Angabe der Versicherungsnummer beim Versicherer (Gerling-Konzern) einzureichen.

Die Adresse lautet: Gerling Industrie Deutschland GmbH

NL West Prinzenallee 21

40549 Düsseldorf

Tel. 0211/ 4956-183

Fax. 0211/ 4956-487

Bitte beachten Sie, dass Ihr Versicherungsschutz bei Nichtbeachten der Versicherungsbedingungen gefährdet ist.

Eine Kopie der Versicherungsbestätigung sowie der allgemeinen Versicherungsbedingungen können Sie über Ihren behandelnden Arzt oder die Studienzentrale erhalten. Die Adresse der Studienzentrale lautet:

Marion Schwartzkopff, Lung Cancer Group Cologne (LCGC), Medizinische Klinik I des Universitätsklinikums Köln, Kerpenerstr. 62, 50924 Köln, Tel.: 0221 – 478 3410, Fax: 0221-478-6733.

Über etwaige Versicherungsleistungen hinausgehende Schadenersatzansprüche sind ausgeschlossen.

Datenschutz

Im Rahmen dieser Studie werden personenbezogene Behandlungsdaten von Ihnen erhoben und an die Studienzentrale in Köln weitergeleitet. Alle Personen, die Einblick in die gespeicherten Daten haben, sind zur Wahrung des Datenschutzes verpflichtet. Ihr Name wird zu keiner Zeit öffentlich gemacht. Die Weitergabe Ihrer Daten erfolgt ausschließlich ohne direkten Bezug zu Ihrem Namen (in der pseudonymisierten Form). Das heißt, alle Dokumente tragen ausschließlich eine Nummer, die keine direkte Verknüpfung mit Ihren persönlichen Daten hat.
Im Rahmen der Überwachung von klinischen Studien können unabhängige Personen (z.B. Monitore) oder Vertreter der Überwachungsbehörden (z.B. Regierungspräsidium, Bundesoberbehörde) Einsicht in Ihre Krankenakten nehmen, um sicherzustellen, dass die Daten in dieser Studie korrekt erhoben wurden. Diese Personen sind jedoch von Amts wegen zur Verschwiegenheit verpflichtet. Mit dem Unterzeichnen der

Einverständniserklärung geben Sie Ihre Einwilligung zur Erhebung und Weitergabe ihrer Daten und Gewebeproben sowie zur Einsichtnahme durch autorisierte Dritte.
Ohne die Einwilligung zur Weitergabe Ihrer pseudonymisierten Daten dürfen Sie in die Studie nicht eingeschlossen werden.

Falls die Studienzentrale über einen längeren Zeitraum keine Informationen von Ihnen erhalten hat, wird sie sich schriftlich (falls sie uns hierzu die Genehmigung erteilt haben) mit der Bitte an Sie wenden, sich zu einer Nachsorgeuntersuchung zu einem Arzt Ihrer Wahl zu begeben bzw. uns die Adresse Ihres behandelnden Arztes mitzuteilen.

Weitere Fragen

Sollten Sie weitere Fragen bezüglich dieser Studie oder Ihren Rechten und Pflichten als Teilnehmer haben, so wenden Sie sich bitte jederzeit an Ihren behandelnden Arzt/Ärztin:

Name: .... Tel..

oder an die Zentrale der Lung Cancer Group Cologne (LCGC)
(Frau Marion Schwartzkopff, Tel.: 0221/478-3410; Fax: 0221-478-6733).

Oder

Für allgemeine Fragen bezüglich klinische Studien
Bundesinstitut für Arzneimittel und Medizinprodukte
Kurt-Georg-Kiesinger-Allee 3
53175 Bonn
Tel.: +49 (0)228 207-30 (Zentrale)
Fax: +49 (0)228 207-5207
E-Mail: poststelle@bfarm.de

Sollten Sie sich für eine Teilnahme an dieser Studie entschließen, möchten wir Ihnen schon jetzt herzlich für Ihre Unterstützung danken.

Einverständniserklärung

zur Teilnahme an

ERLOPET

Eine klinische Studie zur Evaluation von FDG-/FLT-PET zur frühen Vorhersage des Nichtansprechens von Patienten mit der Erstdiagnose eines fortgeschrittenen nicht-kleinzelligen Bronchialkarzinoms auf die Behandlung mit Erlotinib (Tarceva®) und zum Zusammenhang von PET-Befunden und molekularen Markern.

Ich bin von meinem behandelnden Arzt ausführlich und verständlich über die Art meiner Erkrankung, die durchzuführende Behandlung, Wirkungen und Nebenwirkungen, mögliche Spätfolgen und Risiken sowie über Ziele, Meldung, Bedeutung und Tragweite der oben genannten Studie informiert worden. Auf alternative Behandlungsmethoden außerhalb dieses Studienprotokolls wurde ich hingewiesen.
Ich wurde über alle für die Studie notwendigen Untersuchungen und ihre Nebenwirkungen aufgeklärt, insbesondere über die Einspritzung einer schwach radioaktiven Substanz mit ähnlicher Strahlenbelastung wie bei einer CT-Untersuchung. Ich wurde darüber informiert, dass das Medikament Erlotinib (Tarceva®) in Deutschland nicht für die Erstlinienbehandlung des nicht-kleinzelligen Bronchialkarzinoms zugelassen ist und dass die Substanz 18F-Fluorothymidin in Deutschland arzneimittelrechtlich nicht zugelassen ist.
Ich wurde darüber aufgeklärt, dass regelmäßige Untersuchungen in meinem Interesse auch nach abgeschlossener Erlotinib (Tarceva®) Therapie über einen Zeitraum von eineinhalb Jahren durchgeführt werden sollen.
Ich wurde ebenfalls darüber informiert, dass die in dieser Studie eingesetzten Substanzen möglicherweise das ungeborene Leben schädigen können und daher Schwangere von einer Teilnahme ausgeschlossen sind bzw. dass durch sicheren Empfängnisschutz eine Schwangerschaft für die Dauer der Behandlung auszuschließen ist.
Ich wurde über die bestehende Patientenversicherung aufgeklärt sowie über die sich für mich daraus ergebenden Anforderungen. Über die mündliche Aufklärung hinaus habe ich den Text der Patienteninformation und dieser Einwilligungserklärung gelesen und verstanden. Aufgetretene Fragen wurden mir vom behandelnden Arzt verständlich und ausreichend beantwortet. Ein Ansprechpartner für weitere zukünftige Fragen wurde mir genannt.
Folgende Themen wurden zusätzlich im mündlichen Aufklärungsgespräch behandelt:

....
....
....
…………………………………………………………………………………………………

Ich bin damit einverstanden, dass im Rahmen des wissenschaftlichen Projektes DNA-Analysen an den von mir bei der Erstdiagnose entnommenen Gewebeproben

durchgeführt werden und übertrage hiermit der Studienleitung das Verfügungsrecht an diesem Material. Ich verzichte auf ein Entgelt für die Teilnahme an dieser Studie.

Ich bestätige durch meine Unterschrift, dass ich bereit bin, an dem obengenannten Studienprotokoll zur Therapie des fortgeschrittenen Bronchialkarzinoms teilzunehmen. Ich erkläre mich mit der Behandlung gemäß der Studie einschließlich der dafür notwendigen wissenschaftlichen und ärztlichen Untersuchungen einverstanden. Ich bin darüber informiert, dass ich meine heute gegebene Einwilligung jederzeit und ohne Angabe von Gründen widerrufen kann, ohne dass mir daraus Nachteile entstehen.
Eine Kopie dieser Einwilligungserklärung und der dazugehörigen Patienteninformation habe ich erhalten.

Unterschriften:
Aufklärender Arzt

Name:...Unterschrift:.........................Ort/Datum:............................

Patient
Adresse des Patienten:

Straße:..

PLZ/Ort:...

Name:...Unterschrift:.........................Ort/Datum:....................

Zeuge (falls erforderlich)

Name:...Unterschrift:.........................Ort/Datum:................

Ich erkläre mich damit einverstanden, dass ich von der Studienzentrale persönlich angeschrieben werden kann, falls diese über einen längeren Zeitraum keine Informationen von mir erhalten hat. In diesem Fall wird sich die Studienzentrale mit der Bitte an mich wenden, mich bezüglich meiner Untersuchungen zu einem Arzt meiner Wahl zu begeben bzw. ihr die Adresse meines behandelnden Arztes mitzuteilen.

Ort/Datum:……..Unterschrift:.................................

(Patient)

Einwilligungserklärung

in die Erhebung und Verwendung personenbezogener Daten im Rahmen

der ERLOPET- Studie.

Eine klinische Studie zur Evaluation von FDG-/FLT-PET zur frühen Vorhersage des Nichtansprechens von Patienten mit der Erstdiagnose eines fortgeschrittenen nicht-kleinzelligen Bronchialkarzinoms auf die Behandlung mit Erlotinib (Tarceva®) und zum Zusammenhang von PET-Befunden und molekularen Markern.

Ich bin damit einverstanden, dass meine im Rahmen der Studie erhobenen personenbezogenen Behandlungsdaten an die Studienzentrale in Köln weitergeleitet und unter Wahrung des Datenschutzes bearbeitet werden.

Ferner bin ich damit einverstanden, dass im Rahmen der Überwachung von klinischen Studien unabhängige Personen (z.B. Monitore) oder Vertreter der Überwachungsbehörden (z.B. Regierungspräsidium, Bundesoberbehörde) Einsicht in meine Krankenakten nehmen können.

Ich erkläre, dass ich mit der im Rahmen der klinischen Prüfung erfolgenden Aufzeichnung von Krankheitsdaten/Studiendaten und ihrer pseudonymisierten Weitergabe zur Überprüfung an den Auftraggeber, die zuständige Bundesoberbehörde und, soweit es sich um personenbezogene Daten handelt, mit deren Einsichtnahme durch zur Verschwiegenheit verpflichtete Beauftragte des Auftraggebers oder der Behörden einverstanden bin.

Ich bin darüber informiert, dass eine Bezahlung für die Teilnahme an dieser Studie nicht erfolgt.

Ich bin darüber informiert, dass ich an der o.g. klinischen Prüfung nur dann teilnehmen darf, wenn ich der pseudonymisierten Weitergabe meiner Daten zustimme.

Unterschriften:
Aufklärender Arzt

Name:..Unterschrift:........................Ort/Datum:...........................

Patient

Name:..Unterschrift:.........................Ort/Datum:....................

Zeuge (falls erforderlich)

Name:..Unterschrift:........................Ort/Datum:................

Anhang I zur Patienteninformation:

Hinweise zur Anwendung von Tarceva® und Warnhinweise

(Stand Fachinformation 3/06)

Die Tabletten sollten mindestens eine Stunde vor oder zwei Stunden nach einer Mahlzeit eingenommen werden. Ihr Prüfarzt/Ihre Prüfärztin kann ggf. die Dosis anpassen. Für die verschiedenen Dosierungen ist Tarceva® in Stärken von 25 mg, 100 mg oder 150 mg verfügbar. Wird festgestellt, dass bei Ihnen eine schwere Überempfindlichkeit gegen Tarceva® oder einen der sonstigen Bestandteile besteht, darf die Therapie nicht mehr verabreicht werden.

Tarceva® enthält den Zucker Lactose-Monohydrat. Bitte nehmen Sie Tarceva® daher erst nach Rücksprache mit Ihrem Prüfarzt/Ihrer Prüfärztin ein, falls Ihnen bekannt ist, dass Sie unter einer Unverträglichkeit gegenüber bestimmten Zuckern leiden.

Besondere Vorsicht ist bei der Einnahme von Tarceva® und anderen Arzneimittel erforderlich. Es besteht die Möglichkeit, dass Arzneimittel die Konzentration von Tarceva® in Ihrem Blut erhöhen oder erniedrigen und damit die Wirksamkeit von Tarceva® verringern oder die Nebenwirkungen von Tarceva® verstärken können. Für Medikamente zur Blutverdünnung besteht die Möglichkeit, dass durch die Einnahme während der Tarceva® Therapie das Blutungsrisiko verstärkt wird.

Bitte informieren Sie unbedingt Ihren Prüfarzt/Ihre Prüfärztin über alle weiteren Medikamente, die Sie einnehmen und über alle Veränderungen in Ihrem Befinden, da die Behandlung eventuell angepasst werden muss.

Falls Sie Raucher sind, wird Ihnen geraten, das Rauchen einzustellen, da auch Rauchen die Konzentration Ihres Arzneimittels im Blut vermindern kann.

Es ist nicht bekannt, ob Tarceva® eine andere Wirkung hat, wenn Ihre Leber oder Ihre Nieren nicht normal arbeiten. Die Behandlung mit diesem Arzneimittel wird nicht empfohlen, wenn Sie unter einer schweren Leberfunktionsstörung oder einer schweren Nierenfunktionsstörung leiden.

Benachrichtigen Sie Ihren Prüfarzt/Ihre Prüfärztin **umgehend**,

- wenn Sie plötzlich Atemschwierigkeiten bekommen, die mit Husten oder Fieber verbunden sind oder diese unerklärlich lang anhalten. Es besteht die seltene Gefahr, dass bei Ihnen eine so genannte interstitielle Lungenerkrankung, die zum Teil tödlich verlaufen kann, festgestellt wird. Ihr Prüfarzt/Ihre Prüfärztin muss abklären ob bei Ihnen diese spezielle Lungenerkrankung vorliegt. Es könnte sein, dass Ihnen Ihr Prüfarzt/Ihre Prüfärztin andere Arzneimittel verordnen und die Behandlung unterbrechen muss;

- falls Sie an schwerem oder anhaltendem Durchfall, an Übelkeit, Appetitlosigkeit oder Erbrechen leiden; Ihr Prüfarzt/Ihre Prüfärztin wird die Behandlung möglicherweise unterbrechen und geeignete Maßnahmen ergreifen, damit Ihre Körper ausreichend Flüssigkeit hat.

Verkehrstüchtigkeit und das Bedienen von Maschinen:

Es wurde nicht untersucht, ob Tarceva® mögliche Auswirkungen auf die Fähigkeit, Auto zu fahren oder Maschinen zu bedienen, hat. Es ist aber sehr unwahrscheinlich, dass Ihre Behandlung diese Fähigkeit beeinträchtigt.

Risiken und Nebenwirkungen

Tarceva® wurde bisher sicher angewandt bei mehr als 3000 Krebspatienten, die in Phase I, II und III Studien mit Tarceva® allein oder in Kombination mit einer Chemotherapie behandelt wurden. Wie alle Arzneimittel kann die Einnahme von Tarceva® mit Nebenwirkungen verbunden sein.

Als häufigste Nebenwirkungen im Zusammenhang mit einer Tarceva® Therapie wurden Hautausschläge (so genannter Rash) und Durchfälle berichtet. Diese waren größtenteils von leichter Ausprägung und mussten nicht behandelt werden. In einigen Fällen war eine Behandlung notwendig und bei wenigen Patienten musste die Behandlung aufgrund dieser Nebenwirkungen abgebrochen werden. In der Studie BR.21 betrug die Zeitspanne bis zum Auftreten eines Ausschlags etwa 8 Tage und bis zum Auftreten von Durchfall etwa 12 Tage.

Weitere sehr häufig auftretende Nebenwirkungen sind Müdigkeit, Appetitverlust, Atemschwierigkeiten, Husten, Infektionen, Übelkeit, Erbrechen, Reizung der Mundschleimhaut, Magen- und Darmbeschwerden, Juckreiz, trockene Haut, gereizte Augen bzw. Binde- und Hornhautentzündungen. Häufig traten Magen- oder Darmblutungen sowie veränderte Werte für die Leber auf.

Als gelegentliche, schwere Nebenwirkung (aufgetreten bei weniger als 1 von 100 Patienten) kann es zu einer seltenen Form einer Erkrankung der Lungen - einer so genannten

interstitiellen Lungenerkrankung – kommen. Diese Erkrankung kann auch mit dem natürlichen Verlauf Ihrer Erkrankung zusammenhängen und kann in einigen Fällen tödlich verlaufen. Falls bei Ihnen Symptome wie plötzliche Atemschwierigkeiten, verbunden mit Husten oder Fieber, auftreten, **benachrichtigen Sie unverzüglich Ihren Prüfarzt/Ihre Prüfärztin**, da Sie an dieser Erkrankung leiden könnten. Ihr Prüfarzt/Ihre Prüfärztin wird unter Umständen beschließen, Ihre Behandlung mit Tarceva® abzubrechen.

Schwangerschaft/Stillen/Kontrazeption

Schwangere oder stillende Frauen können nicht an dieser Studie teilnehmen, da die Auswirkungen von Tarceva® auf das ungeborene Kind nicht bekannt sind. Obwohl Tarceva® keine genetischen Mutationen oder Chromosomenänderungen (Änderungen am Erbgut) in Zellkulturen verursacht, kann eine Schädigung des ungeborenen Kindes nicht ausgeschlossen werden. Wenn Sie eine Frau im gebärfähigen Alter sind, wird vor Beginn der Studie ein Schwangerschaftstest durchgeführt. Sollte es doch zu einer Schwangerschaft während oder bis zu 90 Tagen nach dem Abschluss der Studie kommen, müssen Sie unverzüglich Ihren Arzt/Ihre Ärztin informieren.

Fruchtbare weibliche und männliche Teilnehmer der Studie müssen eine wirksame Methode der Empfängnisverhütung anwenden. Werden Partnerinnen von männlichen Studienpatienten während der Studie schwanger, so wird man sie bitten, der Firma Roche die Erlaubnis zu erteilen Informationen zum Verlauf der Schwangerschaft einzuholen. In diesem Falle wird man Sie und Ihre Partnerin bitten, hierzu eine gesonderte Einverständniserklärung zu unterzeichnen.

Es ist nicht bekannt, ob Erlotinib in die Muttermilch übergeht. Aufgrund der Gefahr einer Schädigung des Säuglings müssen Mütter angewiesen werden, während der Behandlung mit Tarceva® nicht zu stillen.
